# Macrophage-derived LTB_4_ promotes abscess formation and clearance of Staphylococcus aureus skin infection in mice

**DOI:** 10.1371/journal.ppat.1007244

**Published:** 2018-08-13

**Authors:** Stephanie L. Brandt, Nathan Klopfenstein, Soujuan Wang, Seth Winfree, Brian P. McCarthy, Paul R. Territo, Lloyd Miller, C. Henrique Serezani

**Affiliations:** 1 Indiana University School of Medicine, Department of Microbiology & Immunology, Indianapolis, Indiana, United States of America; 2 Vanderbilt University Medical Center, Department of Medicine, Division of Infectious Disease, Nashville, Tennessee, United States of America; 3 Vanderbilt University Medical Center, Department of Pathology, Microbiology and Immunology, Nashville, Tennessee, United States of America; 4 Indiana Center for Biological Microscopy, Indianapolis, Indiana, United States of America; 5 Indiana Institute for Biomedical Imaging Sciences, Department of Radiology, Indianapolis, Indiana, United States of America; 6 Johns Hopkins University School of Medicine, Department of Dermatology, Baltimore, Maryland, United States of America; 7 Vanderbilt Institute of Infection, Immunology and Inflammation, Nashville, Tennessee, United States of America; Columbia University, UNITED STATES

## Abstract

The early events that shape the innate immune response to restrain pathogens during skin infections remain elusive. Methicillin-resistant *Staphylococcus aureus* (MRSA) infection engages phagocyte chemotaxis, abscess formation, and microbial clearance. Upon infection, neutrophils and monocytes find a gradient of chemoattractants that influence both phagocyte direction and microbial clearance. The bioactive lipid leukotriene B_4_ (LTB_4_) is quickly (seconds to minutes) produced by 5-lipoxygenase (5-LO) and signals through the G protein-coupled receptors LTB4R1 (BLT1) or BLT2 in phagocytes and structural cells. Although it is known that LTB_4_ enhances antimicrobial effector functions *in vitro*, whether prompt LTB_4_ production is required for bacterial clearance and development of an inflammatory milieu necessary for abscess formation to restrain pathogen dissemination is unknown. We found that LTB_4_ is produced in areas near the abscess and BLT1 deficient mice are unable to form an abscess, elicit neutrophil chemotaxis, generation of neutrophil and monocyte chemokines, as well as reactive oxygen species-dependent bacterial clearance. We also found that an ointment containing LTB_4_ synergizes with antibiotics to eliminate MRSA potently. Here, we uncovered a heretofore unknown role of macrophage-derived LTB_4_ in orchestrating the chemoattractant gradient required for abscess formation, while amplifying antimicrobial effector functions.

## Author summary

*Staphylococcus aureus* skin infection is orchestrated by resident and recruited immune cells. The signals that required for the control of *S*. *aureus* infection have been studied. However, most studies have focused on the actions of inflammatory mediators produced after hours post infection and the early events (seconds to minutes) that shape innate immune response is poorly understood. Here, we employed novel techniques, along with epistatic and gain of function experimental procedures, to identify critical components involved in the control of MRSA skin infection. Here, we unveiled the role of skin macrophages in the generation of the lipid mediator leukotriene B_4_ (LTB_4_) that culminates in optimal chemokine/cytokine production, abscess formation, and bacterial clearance. An ointment containing LTB_4_ and antibiotics improved skin host defense and abscess formation and also enhanced bacterial clearance. These results suggest that LTB_4_ could be used locally as an immunotherapeutic agent to strengthen/restore innate immune host activation during MRSA skin infections.

## Introduction

*Staphylococcus aureus* is a significant cause of severe skin and soft tissue infections that can progress to life-threatening infections, such as osteomyelitis or sepsis, if left untreated [1–3]. The inflammatory response to *S*. *aureus* infection is orchestrated by the interaction among structural cells of the skin and both resident and recruited phagocytes [4]. Upon skin infection, keratinocytes and resident immune cells produce antimicrobials to clear the pathogens and generate cytokines, chemokines, and lipid mediators to further activate dermal macrophages and promote neutrophil recruitment to the site of infection [4]. Most studies have focused on the role of cytokines and chemokines as central regulators of skin host defense. Although IL-1β and CXCL2 have been implicated as regulators of neutrophil chemotaxis [5] and IFN-γ and TNFα as potent inducers of phagocyte antimicrobial effector functions [6, 7], the early events that lead to the production of these molecules upon bacterial recognition remain elusive. We and others have shown that the lipid mediator leukotriene B_4_ (LTB_4_) directly enhances neutrophil migration to inflammatory sites and also amplifies *in vitro* pathogen recognition and antimicrobial effector functions [8]. LTB_4_ is produced within seconds to minutes upon phagocyte activation from the multistep metabolism of arachidonic acid (AA) by 5-lipoxygenase (5-LO) to form leukotriene A_4_ (LTA_4_), which is then hydrolyzed to LTB_4_ by LTA_4_ hydrolase [8]. The effects of LTB_4_ are mainly mediated by its high-affinity receptor BLT1 [8], but the low-affinity receptor BLT2 also exerts essential functions in skin homeostasis, cancer, and recruitment of T cells [8, 9]. *In vitro* studies show that LTB_4_/BLT1 also allows TLR activation by increasing MyD88 expression and activating downstream effectors, such as IKK-α and -β, p38 MAPK, IRAK4, and transcription factors such as NFκB, AP-1, and PU.1 [10–13]. We have also shown that aerosolized LTB_4_ increases clearance of lung *Streptococcus pneumoniae*, demonstrating its safety and potential therapeutic application [14]. Furthermore, Yamamoto et al. [15] have shown that treatment of mice with LTB_4_ increased the clearance of MRSA peritoneal infection, but the cellular players and molecular mechanisms involved in *in vivo* host defense are unknown.

A significant component of the host defense during *S*. *aureus* infection is the formation of an abscess. The abscess is composed of bacteria and dead and live neutrophils encapsulated in collagen, fibrin, and other fibrous materials that contain bacteria at the site of infection and prevent dissemination to the bloodstream and other organs [16]. While bacterial products are essential components of a compact and well-organized abscess, the host-derived cellular sources and molecular programs t that promote abscess formation are not well understood [17, 18]. Therefore, therapeutic strategies to improve abscess formation along with enhancing antimicrobial effector functions are highly desirable.

Here, we employed state of the art techniques, along with epistatic and gain of function experimental procedures, to identify critical innate immune components crucial in the abscess formation and control of MRSA skin infection. We showed that while 5-LO^-/-^ and BLT1^-/-^ deficient mice were unable to clear the bacteria and form a well-defined abscess that renders animal more susceptible to skin infection, topical treatment of 5-LO^-/-^ mice with LTB_4_ restored abscess formation and the skin host defenses. We also identified skin macrophages as key players involved in the LTB_4_ production, which were necessary for orchestrating abscess architecture, bacterial clearance, and NADPH oxidase-dependent ROS production, all of which were necessary for abscess formation and MRSA clearance. These findings confirm the concept that endogenously produced LTB_4_ was required for optimal control of MRSA skin infection, an ointment containing LTB_4_ shows synergistic effects with the antibiotic mupirocin to improve skin host defense, abscess formation, while eliminates skin MRSA. These results suggest that LTB_4_ could be used locally as an immunotherapeutic agent to enhance/restore innate immune host activation during antibiotic-resistant bacterial skin infections.

## Results

### MRSA skin infection induces LTB_4_ production in areas near the abscess

**Fig 1:**
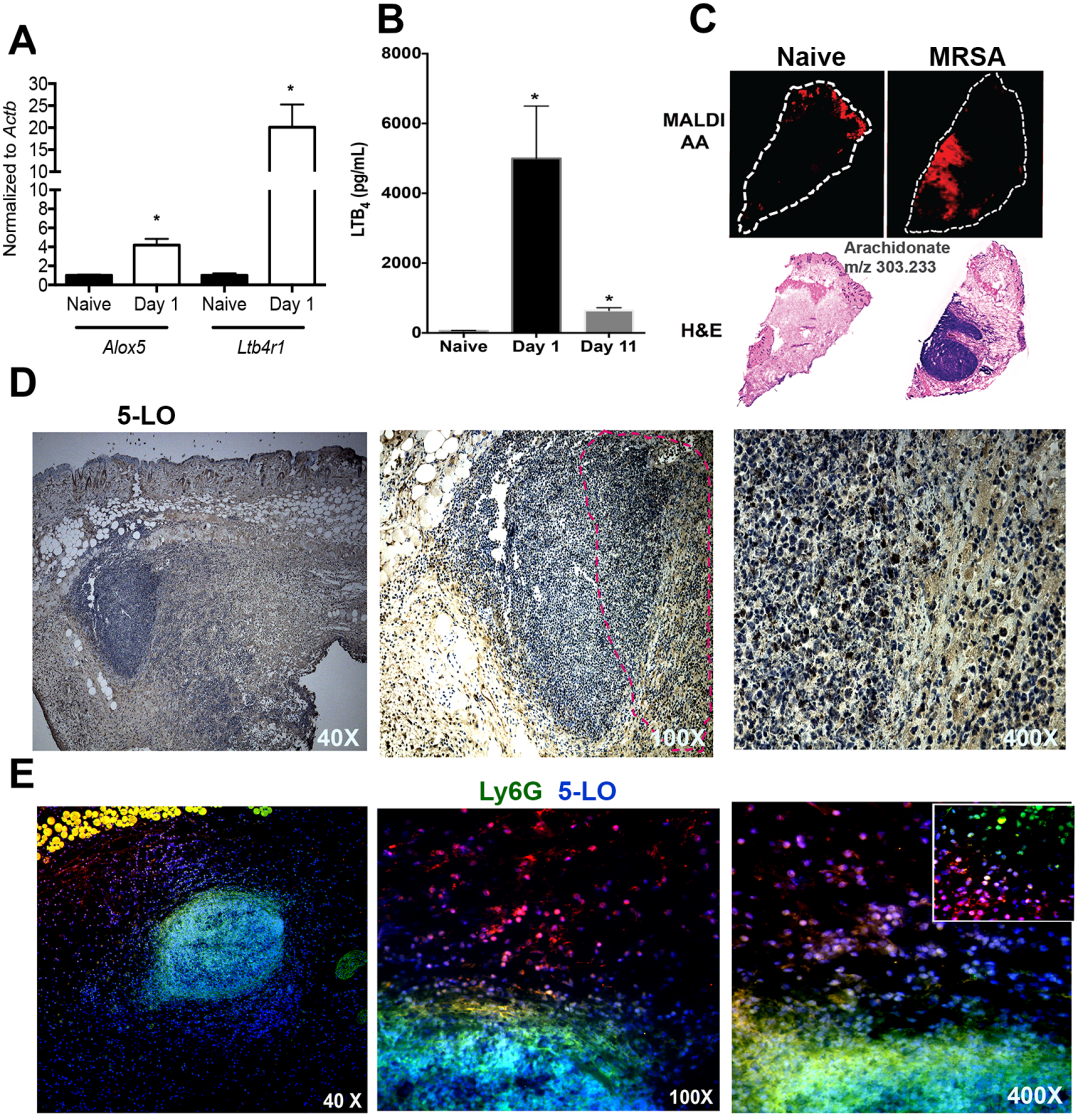
MRSA infection induces LTB_4_ production in areas close to the abscess. **A)** Detection of *Alox5* and *Ltb4r1* from the uninfected naïve skin and day 1 post-MRSA skin infection as assessed by qPCR. **B)** LTB_4_ EIA from skin biopsy homogenates from uninfected naïve samples, days 1 and 11 post-infection. **C)** Top panel: MALDI imaging for AA (red) determined by imaging mass spectrometry as described in the Methods. Bottom panel: H&E stained sections of naïve skin and day 1 post-MRSA skin infection. White dotted line indicates the edge of the skin tissue section. **D)** Expression of 5-LO at 40 X (left panel) and 100 X (middle panel) and 400 X (right) magnification in mice at day 1 after MRSA infection. The 5-LO is shown in brown and counterstained in blue. Data are representative of 2–3 mice. Dotted lines indicate abscess edges. E) Detection of 5-LO (red) and Ly6G (green) in the skin of MRSA-infected mice at 40 X (left panel), 100 X (middle panel) and 400 X (right panel). The inset represents 1000 X amplification. Colocalization between nucleus (DAPI) plus 5-LO is shown as purple, and 5-LO plus Ly6G is shown in yellow. Data are the mean ± SEM from 3–6 mice from 2–3 experiments. *p < 0.05 vs. naïve.

We first measured the mRNA transcript levels for *Alox5* (the gene that encodes 5-LO), *Ltb4r1* (the gene that encodes BLT1), and *Ltb4r2* (BLT2) during MRSA skin infection. At 1 day post-MRSA skin infection, the expressions of *Alox5* and *Ltb4r1*, but not *Ltb4r2*, were significantly enhanced in the skin of infected mice compared to naïve mice skin (Fig 1A). The increased transcripts correlated with higher LTB_4_ production in the skin both early (day 1) and late (day 11) in our study (Fig 1B). Next, we aim to determine the spatial location by which LTB_4_ might influence ski host defense. Using imaging mass spectrometry (IMS), we detected AA mainly on the epidermis in naïve mice. After infection, we identified a robust production of AA in areas near the edge of the abscess (red fluorescence), indicating that LTB_4_ could be produced near the abscess and influence neutrophil migration to the site of infection (Fig 1C). Next, we measured 5-LO expression in the infected skin. Our data clearly showed that 5-LO expression was also localized to areas surrounding the abscess, predominantly in macrophages, and less extensively in neutrophils. We also detected, although in lower amounts, 5-LO staining inside the abscess (Fig 1D). We further confirmed these findings by performing immunofluorescence staining of the infected skin with 5-LO (red) and the neutrophil marker Ly6G (green). We observed that neutrophils express lower abundance of 5-LO than cells with macrophage morphology located surrounding the abscess (Fig 1E). Together these data suggested that endogenously produced LTB_4_ is detected in recruited and activated phagocytes in areas near the abscesses, providing an initial chemoattractant gradient required to mount an efficient host defense during MRSA skin infection.

### LTB_4_/BLT1 is a critical determinant in the outcome of MRSA skin host defense

**Fig 2:**
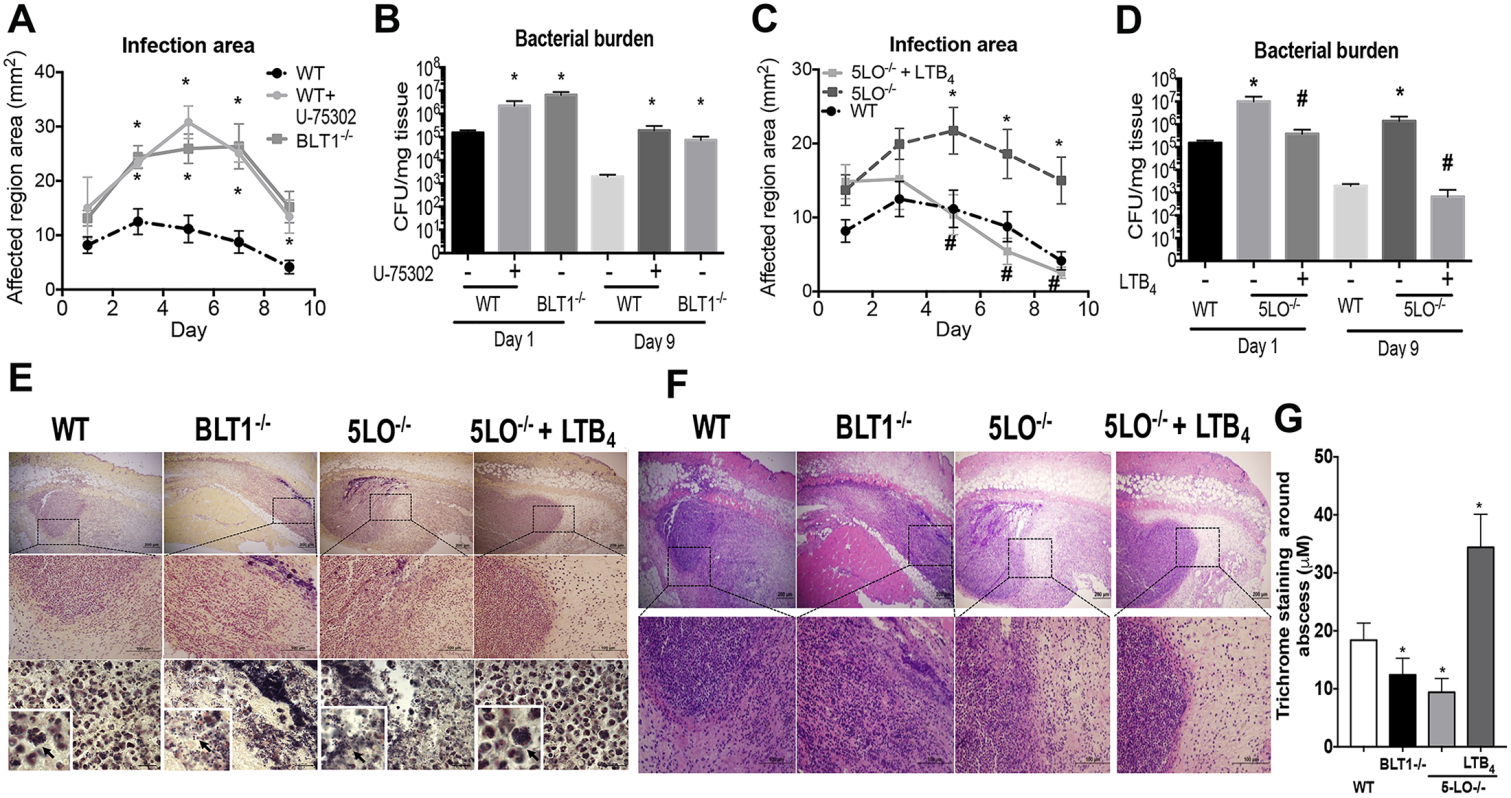
LTB_4_/BLT1 axis is essential to control MRSA skin infections. **A)** The infection area was measured every other day for 9 days in wild-type (WT) C57BL/6 and BLT1^-/-^ mice infected subcutaneously (s.c.) with MRSA and then treated daily or not treated with BLT1 antagonist U75302 (U7) or vehicle control. **B)** Bacterial loads (CFU counts) determined at days 1 and 9 after MRSA skin infection in C57BL/6, 5-LO^-/-^, BLT1^-/-^, and BLT1 antagonist-treated mice. **C**) Infection areas were determined as in (**A**) in the C57BL/6, and 5-LO^-/-^ mice treated or not treated daily with LTB_4_ ointment. **D)** Bacterial load in the infected skin determined as in (**B)** in WT and 5-LO^-/-^ mice treated or not treated with LTB_4_ ointment. **E)** Gram staining of Skin biopsies collected at day 1 post-MRSA skin infection from WT, BLT1^-/-^, 5-LO^-/-^, and 5-LO^-/-^ mice treated topically with 3.37 × 10^−6^% LTB_4_ ointment. Gram staining to label gram-positive bacteria is shown in purple/brown. Top panels are 40 X, middle panels are 100 X, and bottom panels are 400 X magnification Insets represent 1000 X magnification. Black arrows indicate MRSA clusters. Images are representative of 3–5 mice. **F)** H&E stains from mice treated as in (**E)** and shown at 40 X (upper) and 100 X magnification. **G)** Thickness of Masson’s trichrome blue positive layer surrounding the abscess was measured with ImageJ software. In all cases, data represent the mean ± SEM from 3–4 mice. *p < 0.05 vs. WT. ^#^p < 0.05 vs. 5-LO^-/-^ treated with vehicle-control ointment.

Although the role of LTB_4_ in host defense has been explored *in vitro*, most studies did not show the relevance of this bioactive lipid *in vivo*. To that end, mice deficient in LT production (5-LO^-/-^), LTB_4_ responsiveness (BLT1^-/-^) and wild-type (WT) mice were infected with MRSA, and the lesion size and bacterial load studied. As a confirmatory approach, WT mice were treated daily with an ointment containing the BLT1 antagonist, U75302. Genetic or pharmacologic BLT1 deficiency led to increased lesion size and bacterial load at the site of infection (Fig 2A and 2B). Infected 5-LO^-/-^ mice also showed larger infection areas and bacterial burden (Fig 2C and 2D) and adding LTB_4_ back to the 5-LO^-/-^ mice restored the hosts’ skin defenses. Following treatment once daily for 9 days with the LTB_4_ ointment, the 5-LO^-/-^ mice showed reduced infection sizes and the bacterial burdens were comparable to that of the WT mice levels (Fig 2C and 2D). Next, using Gram staining, we observed intracellular bacteria in the WT mice and increased extracellular bacteria in the skin of BLT1^-/-^ and 5-LO^-/-^ mice (Fig 2E). Importantly, treatment of 5-LO^-/-^ mice with LTB_4_ restored engulfment of bacteria (Fig 2E). These data demonstrated that LTB_4_/BLT1 signaling is critical for controlling MRSA skin infections *in vivo* and further showed its therapeutic potential for controlling MRSA skin infections in the absence of LTB_4_.

### LTB_4_ shapes innate immune response during MRSA skin infection

Next, we examined the leukocyte localization and histological changes in the skin of WT, BLT1^-/-^, and 5-LO^-/-^ mice with and without LTB_4_ ointment treatment. Bacterial infections in WT mice resulted in neutrophil and monocyte/macrophage recruitment and abscess formation, as defined by collagen-enriched capsules at the sites of infection (Fig 2F and S1 and S2 Figs). Surprisingly, infections in both BLT1^-/-^ and 5-LO^-/-^ mice led to abundant immune cell recruitment throughout the infected sites, indicating that leukotriene signaling was not required for leukocyte migration to the site of MRSA skin infection (Fig 2F). However, while migrating cells from WT mice developed an organized abscess structure with a fibrous capsule, BLT1^-/-^ and 5-LO^-/-^ infected mice lacked an abscess structure and had poor capsule formation (Fig 2F and S1 and S2 Figs). To further confirm a role for LTB_4_ in abscess formation, 5-LO^-/-^ mice were infected and treated with a topical LTB_4_ ointment. The abscess structures were considerably restored in these mice, suggesting that LTB_4_ was required for normal abscess architecture during MRSA skin infection (Fig 2F and S1 and S2 Figs). Furthermore, we measured the thickness of the capsule, and our data show that while 5-LO^-/-^ mice showed less fibrous capsule surrounding the abscess than infected WT mice, LTB_4_ ointment significantly increased the thickness of the abscess capsule in both WT and 5-LO^-/-^ mice (Fig 2G and S1 and S2 Figs).

Because LTB_4_ is quickly produced, we studied whether blocking LTB_4_ signaling as early as 3 hours after infection will differently influence bacterial clearance and inflammatory response. We observed increased LTB_4_ in the site of infection. When mice were treated with the BLT1 antagonist for 3 hours, we detected higher bacterial load; lower cell infiltrates in BLT1 antagonist-treated mice than mice treated with the vehicle control. Importantly, no changes in CXCL2 and TNF-α between the two experimental groups were identified (S3A–S3E Fig).

To study if lack of abscess is due to uncontrolled bacterial growth in LT-deficient mice, we infected LT-deficient mice with a lower inoculum (5x10^5^ CFU/mL) and our data showed increased bacterial load in 5-LO deficient mice, but did not detect any differences in the production of the inflammatory cytokine TNF-α and, the chemokine CXCL2 when compared to WT control. Interestingly, H&E staining showed that infection of WT mice lead to small swarm formed mainly by polymorphonuclear cells, while the lower number and disorganized phagocytes migration were found in the site of infection in LT-deficient mice (S4A–S4C Fig).

These data show that LTB_4_ production is primarily required for bacterial clearance neutrophil migration and organization in the site of infection which leads to control of bacterial load in the skin.

Next, we determined whether topical LTB_4_ treatment influenced the generation of inflammatory mediators in the skin of infected mice. LTB_4_ treatment increased the production of chemokines (CXCL2, CCL4) and metalloproteinases (MMP8) involved in neutrophil and monocyte recruitment, and inflammatory cytokines (IL-33 and IFN-γ) that are known to enhance macrophage antimicrobial effector functions (S5A–S5D Fig). These data unveiled that LTB_4_ restrains MRSA infection by both controlling phagocyte-mediated bacterial clearance and setting the tone of the inflammatory response that leads to a self-contained abscess formation.

**Fig 3:**
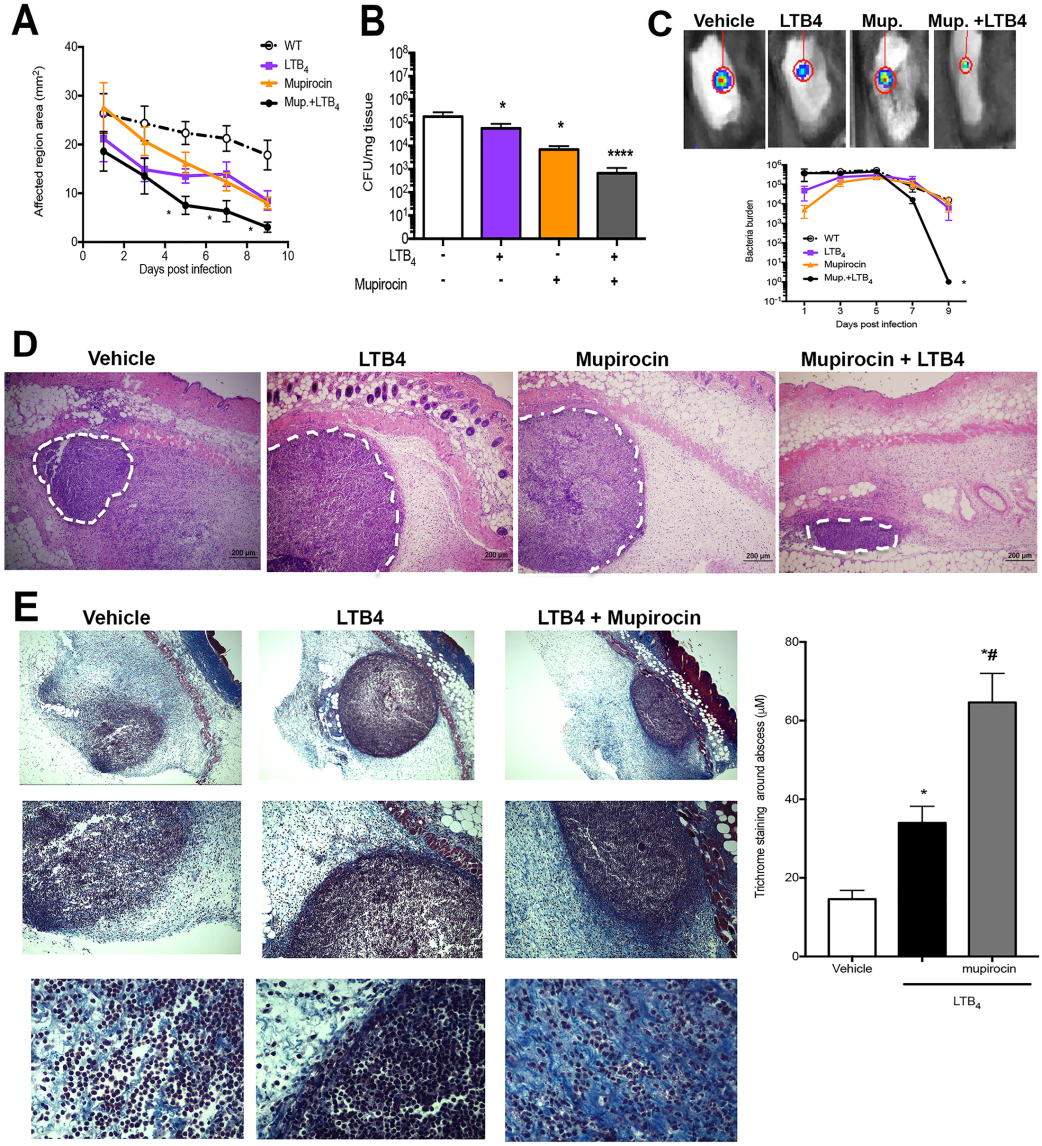
LTB_4_ acts synergistically with mupirocin to increase MRSA clearance. **(A)** Infection area measured every other day for 9 days post infection in WT mice infected with MRSA, s.c., and treated topically daily with vehicle-control ointment, 0.1% mupirocin ointment, LTB_4_ ointment, or mupirocin + LTB_4_ combination ointment. **B)** Bacterial load determined by CFU measured in the skin biopsy homogenates from mice treated as in (**A**) and collected at day 9 post-infection. **C)** Top: Representative bioluminescent MRSA detected in mice treated as in (**A**) using the *in vivo* animal imaging (IVIS Spectrum) detection system for the skin. Bottom: Bacterial load of bioluminescent MRSA in the skin of mice treated as in (**A**) using representative planar bioluminescent imaging (*n* = 5–6 mice/group). **D)** H&E stains from mice treated as in (**A**) and biopsies collected at day 1 post-MRSA skin infection. 40 X magnification and the white dotted lines indicate an abscess edge. **E)** (left) Masson’s trichrome blue staining from mice infected and treated with vehicle, LTB_4_, and LTB_4_ plus mupirocin for 24 h. The top is 40 X, the middle is 100 X and bottom is 400 X magnification. (right) The thickness of Masson’s trichrome blue positive layer surrounding the abscess was measured with ImageJ software as described in the Material and Methods. Data are the mean ± SEM of 4–5 mice from 1–2 experiments. *p < 0.05 vs. vehicle. **** p < 0.001 vs. vehicle.

We then determined whether LTB_4_ synergizes with antibiotics to further improve clearance of MRSA infection. We then employed antibiotic mupirocin, that is effective as a topical ointment. When mice were treated with LTB_4_ plus 0.01% mupirocin, the combination of both agents greatly increased bacterial clearance and decreased lesion size (Fig 3A and 3B). We then determined the time points at which LTB_4_ plus mupirocin were more effective in killing the bacteria *in vivo*. Using a bioluminescent MRSA and monitored bacterial burden over time on the same animal, we observed a highly synergistic effect when mice were treated with both agents (Fig 3C). Furthermore, when we studied the abscess of mice treated as in the Fig 3A, both LTB_4_ and mupirocin alone increased the abscess structure and formed a highly defined abscess than mice treated with the vehicle control (Fig 3D and S1 Fig). The combination therapy further decreased the abscess size and increased the size of the capsule, which correlated with improved bacterial clearance (Fig 3B–3E and S1 Fig). These data show that LTB_4_ is a potent immunotherapeutic agent that influences both early and late events in MRSA infection and synergizes with antibiotic treatment to increase bacterial clearance during MRSA skin infection.

### LTB_4_ shapes the chemoattractant gradient to increase neutrophil directed chemotaxis and increase antimicrobial effectors at the site of MRSA skin infection

**Fig 4:**
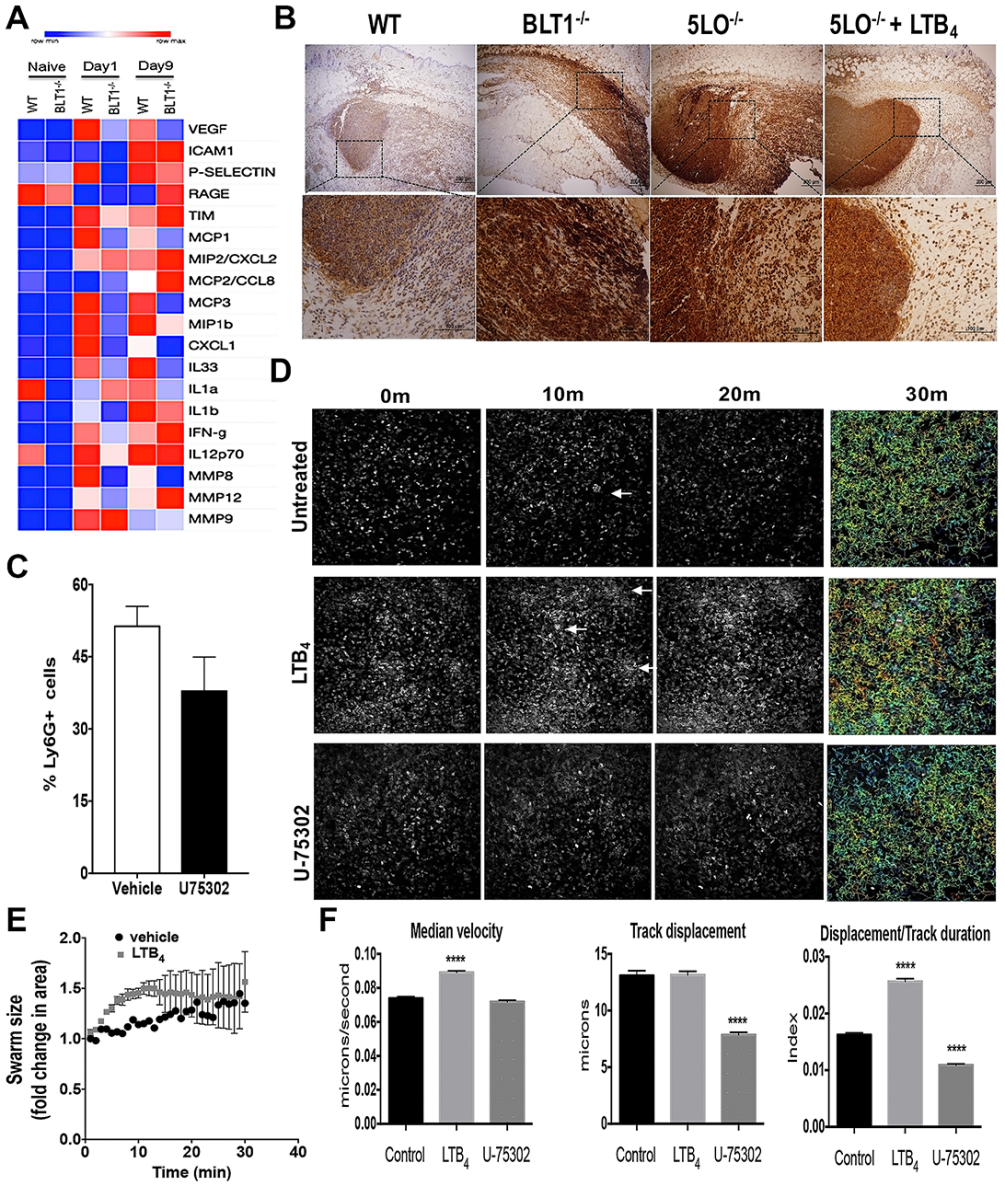
LTB_4_ is required for neutrophil association in the abscess. **(A)** Heat map of proteins involved in inflammatory immune response and resolution in WT and BLT1^-/-^ mice infected with MRSA at day 1 and day 9 after infection measured using bead array multiplex. Proteins are listed on right-hand *y*-axis; grouped alphabetically in clades. Red indicates higher abundance whereas blue represents lower abundance. Each column for each condition represents a technical replicate (*n* = 4-5/group). **B)** Skin biopsy sections from WT, BLT1^-/-^, 5-LO^-/-^, and 5-LO^-/-^ mice + LTB_4_ ointment were stained with anti-Ly6G/C antibody as described in the Material and Methods. Top panels are 40 X, and bottom panels are 400 X magnification. Neutrophil staining is shown in brown and counterstained in blue. **C)** Detection of Ly6G positive cells in the skin biopsies of mice infected and treated with the vehicle control or the ointment containing 0.001% BLT1 antagonist for 24 h using FACS analysis. **D)** Still frames of the intravital imaging from the EGFP-LysM mice infected with MRSA and treated with ointments containing vehicle control (untreated), 3.37 ng LTB_4_ ointment, or 0.001% BLT1 antagonist (U75302). After 24 hours, the mice were imaged by two-photon intravital microscopy for 30 minutes. **E)** Determination of the swarm size in WT EGFP-LysM mice treated or not with the LTB4 ointment using image J as described in the Material and Methods. **F)** Quantifications and track paths of mice represented in (**C**) that show the median velocity of the GFP^+^ cells. Track displacements of GFP^+^ cells and ratios of displacement/track durations were measured using FIJI (ImageJ) Trackpath plugin software and analyses were as described in the Material and Methods. Data are the mean ± SEM of 1000+ GFP^+^ cells representative of 3–4 mice. ****p < 0.0001 vs. untreated. White arrows indicate swarming.

To determine the step(s) of LTB_4_ contribution to the increased host defense during skin infection, we assessed the production of inflammatory cytokines, chemokines, and molecules involved in tissue destruction at early (day 1) and late (day 9) time points after infection in both WT and BLT1^-/-^ mice. Our data (Fig 4A) clearly showed that at day 1 after infection, we detected high levels of chemokines involved in both neutrophils and monocyte recruitment to the site of infection (CXCL2, CXCL1, CCL8, CCL4, CCL2, and CXCL1), along with cytokines known to increase macrophage and neutrophil antimicrobial effector functions, such as IFN-γ and IL-12p70 in WT mice (Fig 4A). In contrast, these inflammatory mediators were decreased in BLT1^-/-^ mice at day 1 after infection. However, BLT1^-/-^ mice had increased levels of the major neutrophil chemoattractant CXCL2, possibly explaining the increased neutrophil numbers in these animals. When the infection was mostly cleared in WT mice (day 9), we observed a decrease in most chemokines, but not in IL-1α and IL-1β, CCL7, and CCL4. In BLT1^-/-^ mice, the inflammatory process was still intense as characterized by higher levels of RAGE, TIM, CXCL2, IFN-γ, MMP12, and CCL8. At this time point, we also detected decreased VEGF, P-Selectin, CCL2, CCL7, CCL4, IL-1α, IL-1β, and IL-33 in BLT1^-/-^ when compared to that in the infected WT mice (Fig 4A). These data suggested that BLT1 was essential in controlling early events involved in the recruitment of effector cells during infection, and lack of BLT1 led to chronic inflammatory responses, as characterized by increased inflammatory cytokines chemokines and danger-associated molecular pattern, such as RAGE (Fig 4A).

Next, we stained the infected skin of WT, BLT1^-/-^, and 5-LO^-/-^ mice using anti-Ly6G/C antibodies to further study whether LTB_4_ controls neutrophil localization. Our data showed that in WT mice, neutrophils were located mainly in the abscess and surrounding the abscess area. However, neutrophils from BLT1^-/-^ and 5-LO^-/-^ mice were not confined to an abscess structure, with the cells found in all layers of the dermis (Fig 4B). Importantly, treatment of 5-LO^-/-^ mice with topical LTB_4_ ointment restored abscess formation and neutrophil location inside the abscess (Fig 4B). We also confirmed that blocking BLT1 decreases neutrophil migration to the site of infection (Fig 4C).

Next, we sought to investigate how LTB_4_ affected neutrophil directed chemotaxis in the infected skin using intravital microscopy imaging. GFP-expressing myeloid cells (LysGFP) mice, were infected with MRSA and treated with the LTB4 or BLT1 antagonist ointment. While neutrophils (brighter GFP signals than other myeloid cells [19] in the skin of mice treated with vehicle ointment formed swarm-like clusters (Fig 4C and 4D), mice treated with topical LTB_4_ formed more neutrophil swarm-like clusters than was shown in the vehicle-treated mice (Fig 4C and 4D). However, BLT1 antagonist treatment did not decrease neutrophil accumulation after 24 h of infection. Additionally, although BLT1 antagonism did not affect cellular velocity, the neutrophil median velocity was increased when mice were treated with LTB_4_ (Fig 4E and 4F). The displacement values were similar between the LTB_4_-treated and untreated control mice. However, BLT1 antagonism significantly reduced the movement of neutrophils in the infected mice (Fig 4C). The ratio of displacement (vector of distance from point A to point B) to duration was used to estimate the cellular directionality and our data showed that the ratio of displacement/duration was increased with LTB_4_ treatment, and decreased with BLT1 antagonist treatment (Fig 4F).

Here we demonstrated that early LTB_4_ production and BLT1 activation dictates skin host defense by controlling both neutrophil chemotaxis and the generation of antimicrobial effector during MRSA skin infection.

### Cross-talk between tissue macrophages and LTB_4_ in MRSA skin infection

**Fig 5:**
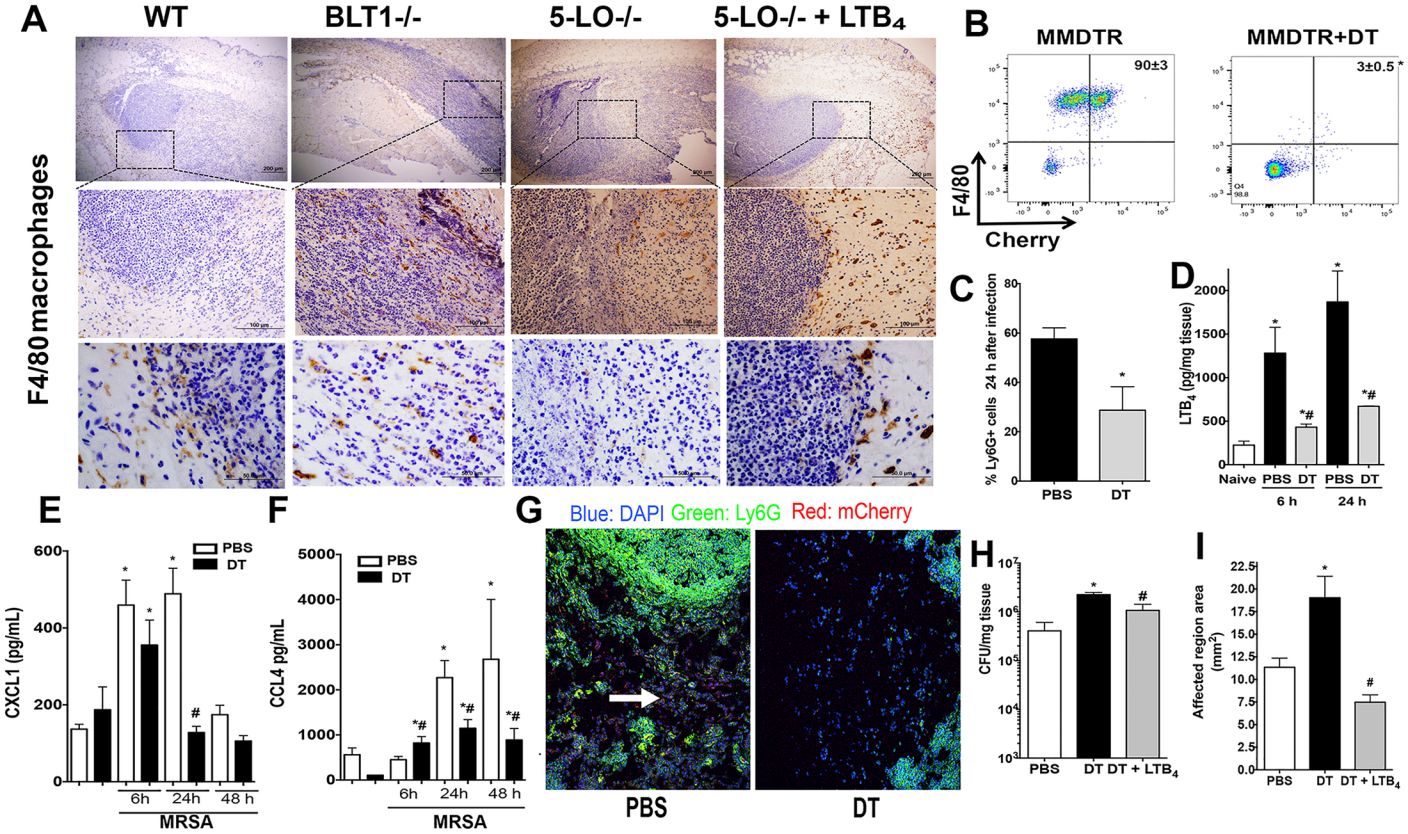
Skin macrophages are necessary for LTB_4_ production and host defenses during MRSA skin infection. **A)** Skin biopsy sections from WT, BLT1^-/-^, 5-LO^-/-^, and 5-LO^-/-^ plus the LTB_4_ ointment treated mice stained with the anti-F4/80 antibody as described in Material and Methods. The top panels are 40 X, the middle panels are 400 X, and the bottom panels are 1000 X magnification. Macrophages stained brown, and the counterstain is blue. Black arrows indicate macrophages and the black dashed box is the amplified region. Images are representative of 3–5 mice. **B)** Depletion of skin macrophages in MMDTR mice were PBS-treated or DT-treated and infected with MRSA by subcutaneous injection. **C)** Percentages of Ly6G^+^ cells in MMDTR mice treated or not treated with DT, followed by MRSA infection for 24 hours, and examined by FACS analyses. **D)** LTB_4_ production in the skin of MMDTR treated or not treated with DT mice, followed by infection at the indicated time points as detected by EIA. **E and F)** Production of CXCL1 **(E)** and CCL4 **(F)** in the skin biopsies from MMDTR mice treated or not treated with DT, followed by MRSA skin infection for the indicated time points as detected by ELISA. **G)** Slides were stained using DAPI (blue), mCherry for macrophages (red), and FITC-labeled anti-Ly6G antibody (green). Images shown are at 200 X magnification and are representative of 3–5 mice/group. White arrows indicate mCherry^+^ stained macrophages. **H)** Bacterial loads of MMDTR mice treated or not treated with DT, infected with MRSA, and treated with LTB4 ointment for 24 hours as measured by CFU counts/mg tissue. **I)** Lesion sizes of MMDTR mice treated as in (**F**) and measured 24 hours after infection. Data are the mean ± SEM of 3–8 mice. *p < 0.05 vs. naïve. ^#^p < 0.05 vs PBS-treated mice.

Next, we aimed to identify whether macrophage localization and actions in the abscess could influence the production of inflammatory mediators and abscess formation during skin infection. In WT mice, macrophages were found along the periphery of the abscess and within the fibrous capsule (Fig 5A). However, macrophages were randomly distributed throughout the areas of the dermis and in the in the central areas of the infectious foci in the BLT1^-/-^ and 5-LO^-/-^ mice (Fig 5A). We then determined whether LTB_4_ restored macrophage localization to areas near the abscess seen in WT mice. Our data clearly show that topical LTB_4_ restored macrophage localization to the periphery of the abscess in 5-LO^-/-^ mice (Fig 5A). Furthermore, these data led us to speculate that macrophage LTB_4_ production near the abscess is necessary for neutrophil recruitment to the abscess.

**Fig 6:**
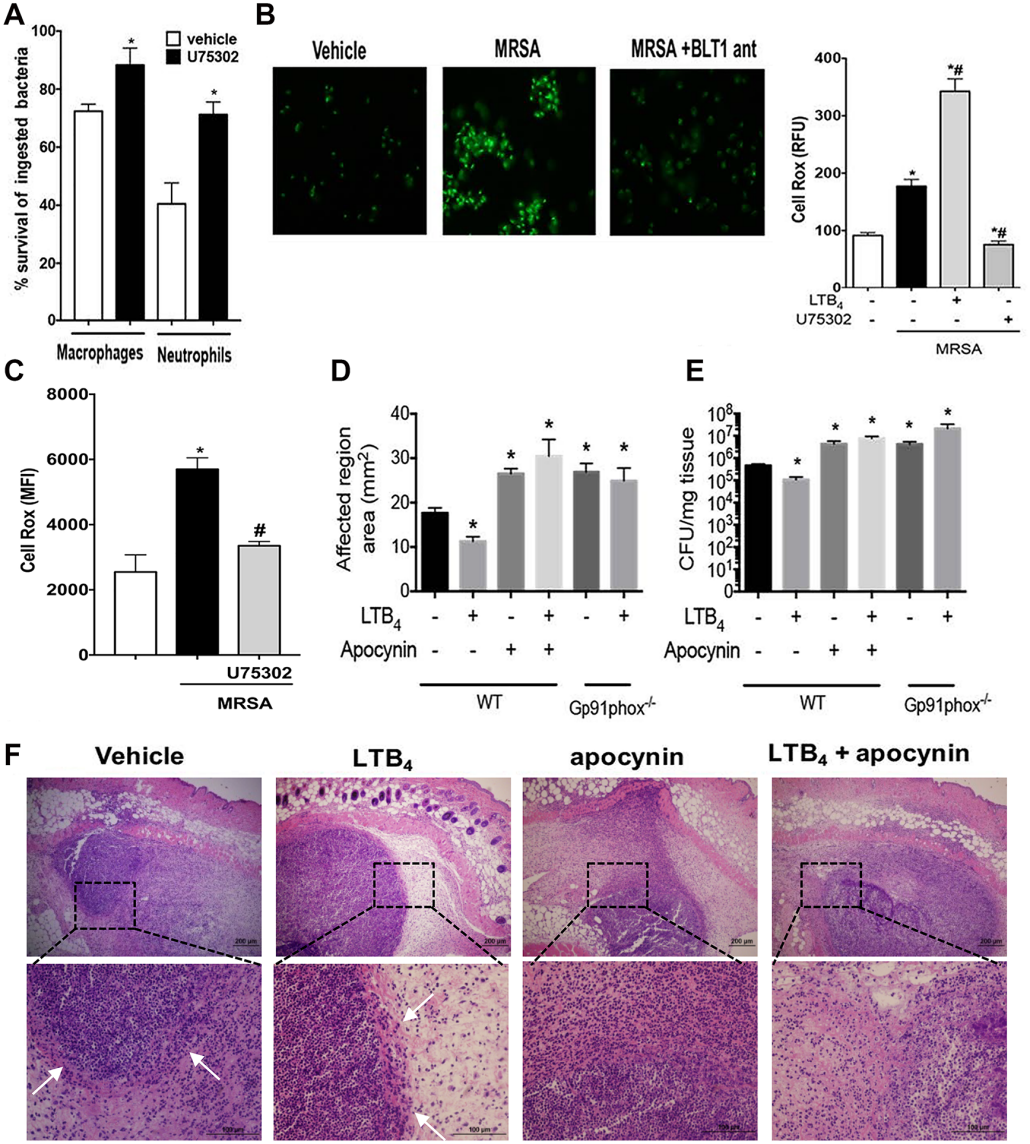
LTB_4_ promotes NADPH oxidase-mediated killing of MRSA. **A)** Determination of bacterial killing in peritoneal macrophages and bone marrow-derived neutrophils from WT and BLT1^-/-^ mice as described in the Material and Methods. **B)** (left) Determination of reactive oxygen species (ROS) production in macrophages from WT mice treated or not treated with the BLT1 antagonist, followed by challenge with MRSA for 60 minutes. (Right) Relative fluorescence units (RFU) levels of CellRox positive macrophages treated with vehicle control, LTB_4_, and BLT1 antagonist, followed by MRSA infection for 60 minutes. The RFU were determined as described in the Material and Methods. **C)** Mean fluorescence intensity (MFI) levels of CellRox positive bone marrow-derived neutrophils treated with vehicle control and BLT1 antagonist, followed by MRSA infection for 60 minutes. The MFI was determined as described in the Material and Methods. **D)** WT or gp91phox^-/-^ mice were infected with MRSA via s.c. and treated topically daily with vehicle-control ointment, apocynin, LTB_4_ ointment, or apocynin + LTB_4_ combination ointment. Infection areas were measured 24 hours after infection, and the affected areas were measured as described in the Material and Methods. **E)** Bacterial loads of WT and Gp91phox^-/-^ mice treated as in (**D**) and CFU were determined 24 hours after infection as described in the Material and Methods. **F)** H&E staining of mice treated and infected as in **(D)**. The top panels are 4 X, and the bottom panels are 40 X magnification. Data are the mean ± SEM of 8–15 mice. *p < 0.05 vs. WT. White arrows indicate abscess edges. All abbreviations are defined in Figs 1 and 2 legends.

We then determined if depletion of skin macrophages influenced the *in vivo* LTB_4_ production, neutrophil recruitment, and abscess formation during MRSA skin infection. Confirming the depletion studies, the diphtheria toxin (DT) treated mice (S5A Fig) exhibited lower mCherry fluorescence as detected using IVIS SpectrumCT *in vivo* imaging and FACS before infection and at day 1 post-infection (S6B Fig and Fig 6B). Depletion of macrophages led to both decreased neutrophil migration and LTB_4_ production at 6 and 24 hours after skin infection (Fig 5B and 5C). In addition, macrophage depletion led to reduced production of CXCL1, and CCL4 24 hours after infection (Fig 5E). Next, we hypothesized that macrophage depletion could compromise abscess structure, as observed in the 5-LO^-/-^ or BLT1^-/-^ mice. Confocal microscopy imaging of infected MMDTR mice showed an organized abscess structure at day 1 post-infection that was composed predominantly of neutrophils, as indicated by Ly6G+ staining (green), and was surrounded by mCherry^+^ macrophages (red) (Fig 5F), supporting our observations using immunohistochemistry (IHC) staining as shown in Fig 5A. DT treatment of MMDTR mice led to a reduction in neutrophils recruited to the skin and abscess formation (Fig 5H), which correlated with worse infection areas and higher bacterial burdens (Fig 5G and 5H) when compared to that of the WT mice treated with DT. These results suggested that macrophages played a central role in LTB_4_ production during MRSA skin infections, which was critical for abscess formation and bacterial clearance. To confirm that LTB_4_ was involved in poor host defenses in macrophage-depleted cells, DT-treated MMDTR were subjected to the LTB_4_ ointment for 24 hours. Our results showed that topical LTB_4_ decreased lesion size and bacterial clearance in macrophage-depleted mice (Fig 5G and 5H), restored production of the chemokines, CCL2 and CXCL4, but not CXCL2 (S7A–S7C Fig), VEGF, IL-33, and IL-1β (S7D–S7F Fig) and neutrophil migration to the site of infection (S7G Fig) in DT-treated MMDTR mice. Together, these findings identified a novel role for skin resident macrophages in regulating inflammatory response and abscess formation during MRSA skin infection.

### MRSA clearance requires LTB_4_-mediated NADPH oxidase activity in vivo

The generation of ROS is essential in the control of *S*. *aureus* infection [16, 20]. LTB_4_ utilizes NADPH oxidase to increase in vitro *Klebsiella pneumoniae* killing. The role of LTB_4_-mediated NADPH oxidase activity in killing MRSA *in vivo* was determined employing both pharmacological and genetic approaches. Initially, we determined whether LTB_4_ is required for MRSA-induced bacterial killing in both macrophages and neutrophils. Our data showed that blocking BLT1 signaling decreases bacterial killing in both cells (Fig 6A), which correlated with decreased ROS production in MRSA-challenged phagocytes pretreated with the BLT1 antagonist (Fig 6B and 6C). Next, we tested whether LTB_4_ enhances bacterial clearance *in vivo*. WT mice were treated with topical ointments containing LTB_4_, an NADPH oxidase inhibitor, apocynin [21], or a combination of both agents after the MRSA infection. Furthermore, The WT mice and the gp91phox^-/-^ mice were treated daily with the LTB_4_ ointment. Here, we observed higher lesion size and bacterial loads when NADPH oxidase was deficient, and while LTB_4_ alone increased bacterial clearance in WT mice, LTB_4_ treatment did not reduce the infection areas or bacterial burdens, and both the apocynin co-treatment or gp91phox^-/-^ mice showed similar results, further confirming that LTB_4_ increased bacterial killing by activating NADPH oxidase *in vivo* (Fig 6D and 6E). We then determined whether ROS influenced abscess formation and if LTB_4_-mediated abscess formation was dependent on ROS production. The WT mice treated with apocynin alone showed poor abscess structure, and LTB_4_ co-treatment with apocynin failed to restore abscess morphology, correlating with higher bacterial burdens (Fig 6F and S1 Fig). These results demonstrated that formation of the abscess and bacterial clearance were dependent upon LTB_4_-dependent NADPH oxidase activities.

## Discussion

MRSA, previously a nosocomial pathogen, has reached epidemic proportions and now is commonly found in both community and hospital settings [22, 23]. Infections with antibiotic-resistant pathogens significantly limit treatment options and could lead to irreversible tissue damage and co-morbidities associated with chronic infections [24]. The development of host-derived immunotherapeutics that boost innate immune response and limit antibiotic resistance to avoid alterations in microbiome populations is much needed. Therefore, combination therapies using endogenous molecules along with the use of antibiotics represent a new frontier in the control of antibiotic-resistant pathogens [25].

**Fig 7:**
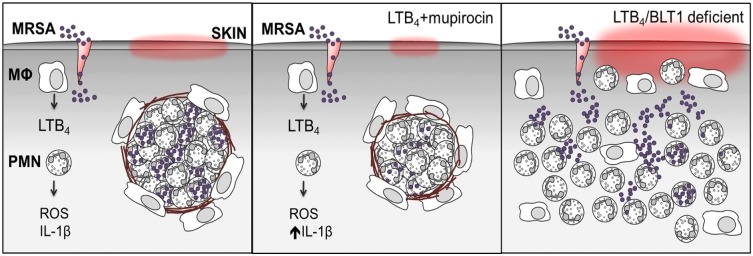
Summary of LTB_4_-promoted MRSA skin infection clearance. Left panel: MRSA skin infection requires tissue-resident macrophages for optimal LTB_4_ production and neutrophil recruitment. LTB_4_ provides direction to neutrophils to contain bacteria and form an abscess surrounded by fibrous collagen. Middle panel: Combination ointment with LTB_4_ + the topical antibiotic, mupirocin, is more efficient at eliminating the infection. Right panel: Mice lacking LTB_4_/BLT1 fail to contain bacteria or develop an abscess. All abbreviations are defined in Figs 1 and 2 legends.

Among the major cellular players involved in MRSA control, keratinocytes, macrophages and neutrophils are essential in recognizing and eliciting bacterial clearance by producing inflammatory mediators that create chemoattractant gradients required for the recruitment of immune cells to the site of infection, acting in an autocrine fashion to increase leukocyte action, and enhance the generation of microbicidal molecules [4]. Consequently, pleiotropic endogenous molecules that act on amplifying both inflammatory response and antimicrobial effector functions are, in theory, optimal immunotherapeutics to treat antibiotic-resistant infections. Furthermore, the earlier events that shape innate immune response during skin infection is not well appreciated. Here, we focused our efforts in understanding the role of a lipid mediator (LTB_4_) that is produced within seconds to minutes after microbial challenge and could dictate the outcome of infection. LTB_4_ has been shown to improve phagocyte effector functions *in vitro* [14, 26–32]. Here, by employing a variety of epistatic and gain of function experiments, we unveiled that LTB_4_ is required to control different steps of the inflammatory response that culminates in a well-capsuled abscess that retains and control MRSA skin infection and that topical LTB_4_ treatment synergizes with the over-the-counter antibiotic mupirocin that greatly improves abscess formation and accelerates bacterial clearance (Fig 7). In summary, we found that: 1) LTB_4_ production in areas near the abscess sets the stage for the neutrophil and macrophage chemotaxis during MRSA skin infection. 2) Endogenously LTB_4_ production is required for host control during MRSA skin host by chemotaxis in the tissue, bacterial killing (ROS production) and inflammatory response (chemokines and cytokines) that directly regulates abscess architecture and bacterial control. 3) Skin macrophages govern neutrophil migration, abscess formation, and bacterial clearance.

Although the role of proteins and lipids in inflammatory processes are studied using strategies to either inhibit or overexpress a given molecule, the exact location of inflammatory mediators in the site of infection is poorly understood. Here we show that LTB_4_ production in areas near the edge of the abscess (as evidenced by enriched 5-LO staining in macrophages and less abundant in neutrophils and AA detection determined by MALDI-IMS) might directly influence leukocyte behavior and increase antimicrobial effector functions. These findings unlock new biology, since identifying the location of inflammatory mediators during infections could uncover regulatory events necessary for optimal host defense *in vivo*. LTB_4_ production in areas near the abscess could favor directed chemotaxis of neutrophils to the site of infection and shape production of inflammatory mediators to elicit phagocyte antimicrobial effector functions.

*S*. *aureus* abscess is molded by several layers of neutrophils, dead cells and macrophages, surrounded by a capsule of fibrin/collagen that contains the pathogen [16, 17]. Although a number of the pathogen-derived virulence factors and cell wall components required for abscess formation have been studied [17, 33], less is known about host-derived products produced during *S*. *aureus* infection and how endogenous inflammatory mediators influence the pathogenesis and abscess formation during staphylococcal infections. Here, genetic and pharmacologic approaches unveiled that LTB_4_ deficiency did not impact neutrophil migration to the site of infection. While MRSA infection led to the formation of a neutrophilic self-confined abscess in WT mice, neutrophils in 5-LO^-/-^ and BLT1^-/-^ infected mice were found within all layers of the dermis and near the epidermis. Importantly, topical LTB_4_ treatment in 5-LO^-/-^ mice restored abscess formation, which indicates that LTB_4_ provides neutrophils cues to locate the abscess (see below). Importantly, poor abscess formation could also be due to an excessive bacterial growth that could delay abscess formation in the abscess of LTB_4_, but when we infected WT and 5-LO^-/-^ mice with 100 times less bacteria than we used in this study, we detected a small neutrophilic swarm in WT but not in LT-deficient mice. The fact that LTB_4_ restores abscess and bacterial clearance in 5-LO^-/-^ is of importance, since acquired states of LT deficiency, such as bone marrow transplant, protein-calorie malnutrition, HIV infection that are highly susceptible to *S*. *aureus* infection are also characterized by low 5-LO expression and LTB_4_ production [8]. Therefore, using LTB_4_ to boost host defense in these groups of patients can be an attractive immunotherapeutic strategy to control infection.

We then determined whether LTB_4_ could play a differential role in different steps of the *S*. *aureus* abscess formation and clearance. Initially, we studied whether blocking LTB_4_ early in the infection could influence bacterial clearance, neutrophil migration and production of inflammatory molecules. Our data show that LTB_4_ is produced after 3 hours of infection, and BLT1 blockage increased bacterial loads and leukocyte migration but did not influence cytokine and chemokine production. These data suggest an essential role for LTB_4_ in the establishment of the skin infection. Next, we determined whether LTB_4_ would alter the synthesis of chemokines and cytokines involved in neutrophil and monocyte chemotaxis during *S*. *aureus* skin infection in WT and BLT1^-/-^ at the beginning of the infection (day 1) and when the lesion is cleared in WT mice (day 9). LTB_4_/BLT1 axis is required for the synthesis of chemokines that recruits neutrophils (CXCL1 and CXCL2), monocytes (CCL2, CCL8, and CCL7) and T cells (CCL4) at both days 1 and 9 after infection. However, CXCL2 levels were substantially increased in infected BLT1^-/-^, which could explain why initial neutrophil migration is not affected in these mice. Given the differential regulation of chemokines in BLT1^-/-^, we speculate that increased CXCL2 is not a compensatory mechanism. Considering that infected BLT1^-/-^ still shows enhanced metalloprotease MMP12, the alarmin RAGE, IFN-γ and decreased VEGF suggest an active inflammatory process, due to inefficient bacterial clearance. Although, BLT1^-/-^ neutrophils were capable of migrating to the site of injury, indicates that LTB_4_/BLT1 does not influence the steps involved in neutrophil diapedesis, such as rolling, adherence and transmigration [34]. LTB_4_ has been shown to act as a signal-relay signal promoting enhanced neutrophil migration towards other chemotactic molecules such as Fmlp [35]. However, the ability of BLT1^-/-^ to be directed to a focal point in the skin was impaired as shown in both our MRSA infection model as well as in a sterile injury model [36], suggesting LTB_4_ functions to provide direction towards a chemoattractant, which is necessary to eliminate infectious products or dead tissue. This phenomenon was recently termed neutrophil swarming [36]. It remains to be determined what are the intracellular events triggered by LTB_4_/BLT1 that provides neutrophil swarming. Here, our data suggest that LTB_4_ production is required during different phases of the infection, namely the initial recruitment of phagocytes to control infection and when the infection is established by locally amplifying antimicrobial effector functions, as well as the inflammatory milieu. Furthermore, we are providing new evidence that topical ointment containing LTB_4_ indeed increases neutrophil swarming during MRSA infection, in addition to increased velocity and neutrophil displacement.

During the initial hours after infection, it has been suggested that skin-resident cells are responsible for neutrophil recruitment to the site of infection [37–39]. However, the specific role of macrophages in neutrophil migration during skin infection remains to be fully determined. Here, using an animal model that specifically depletes macrophages and monocytes, we unveiled that skin macrophages are required for critical events in MRSA skin infection, namely LTB_4_ production and neutrophil migration to the site of infection, abscess formation, and bacterial clearance. Although we depleted cells before infection, we cannot rule out whether recruited monocytes are also crucial for initial LTB_4_ production and neutrophil recruitment. Since we detected these differences as early as 6 hours after infection, we anticipate that skin resident macrophages are dictating early immune response during MRSA skin infection. Also, Feuerstein et al. suggested that MyD88 expression in macrophages is also required for abscess formation. However, the authors used liposomal clodronate that depletes several phagocytes [40]. Interestingly, we have shown that IL-1β/MyD88 actions in neutrophils are necessary for *S*. *aureus* abscess formation [41]. Furthermore, we have previously demonstrated LTB_4_ increases MyD88 expression in macrophages from different organs [10–12]. Whether LTB_4_/MyD88 axis regulates abscess formation during MRSA skin infection remains to be investigated.

Given the fact that LTB_4_ exhibits pleiotropic effects during MRSA skin infection, a topical ointment containing LTB_4_ is a promising therapeutic strategy for treating MRSA skin infections. Interestingly, LTB_4_ enhances the killing of different pathogens by inducing ROS, RNI and antimicrobial peptides *in vitro* [14, 28, 42]. We also have shown that treatment of WT mice with bestatin (a nonspecific LTA_4_ hydrolase and aminopeptidase inhibitor [43]) decreases neutrophil migration and increases bacterial load in after days 1 and 3 after infection [44]. However, the use of bestatin, which also inhibits di-peptidases with inflammatory actions, could potentially influence skin host defense in a manner independent of LTB_4_ production [43, 45]. Yamamoto et al. have shown that LTB_4_ injection increases MRSA clearance and animal survival in a model of peritonitis [15]. Here, we are showing that LTB_4_ activation of NADPH oxidase is required for microbial clearance and improve lesion during MRSA infection. Furthermore, we observed that topical treatment with an ointment containing LTB_4_ and the topical antibiotic mupirocin quickly reduced bacterial burden and infection size. We used mupirocin because it is a topical antibiotic used to treat skin infections and only functions locally. There are many potential advantages to using LTB_4_ in immunotherapeutic protocols as follows: (1) LTB_4_ synthesis is easily produced with a high degree of purity; (2) LTB_4_ has a short half-life, which allows precision in controlling undesired inflammatory response; (3) LTB_4_ is safe to humans and nonhumane primates [42, 46, 47]; and (4) LTB_4_ is a pleiotropic molecule that amplifies initial anti-MRSA responses by enhancing bacterial recognition and phagocytosis [12, 48, 49], release of ROS [49], IL-1β levels, and pro-inflammatory responses through MyD88 expression and NFκB activation [49] that culminates in efficient abscess formation.

In our preclinical studies, we have identified a previously unknown role of tissue-resident macrophages to promote LTB_4_ production, which is necessary to enhance structured abscess formation and bacterial clearance. These studies have evident translational importance given the prevalence of skin infections in patients with immunosuppressive diseases that exhibit poor LTB_4_ production, such as cancer and malnutrition [8]. Therefore, a therapeutic intervention of these groups of patients with an ointment containing LTB_4_ with concurrent antibiotic therapy is a promising strategy to treat MRSA infections.

## Methods

### Study design

For all experiments, the minimum sample size was determined to detect a difference between group means of two times the observed standard error (SE), with a power of 0.8 and a significance level of 0.05, using the power and sample size calculator (http://www.statisticssolutions.com/). On the basis of this, the calculated minimum sample sizes ranged from three to four depending on the experiment. The average sample size for mouse studies was five per group. All samples were randomized but not blinded.

### Animals

Eight—ten-week-old female or male 5-LO^-/-^ (B6.129-*Alox5*^*tm1Fun*^; [50]), BLT1^-/-^ (B6.129S4-*Ltb4r1*^*tm1Adl*^/J;[51], Csf1r-HBEGF/mCherry)1Mnz/J (JAX stock #024046) [52], LysMcre, MMDTR, and strain-matched WT C57BL/6 mice were purchased from Jackson Labs (Bar Harbor, ME USA). MMDTR mice were generated by breeding the Csf1r-HBEGF/mCherry)1Mnz/J plus LysMcre mice as previously reported [52]. EGFP-LysM was donated by Dr. Nadia Carlesso (City of Hope, Duarte, CA, USA), and the pIL1DsRed (donated by Dr. Akiko Takashima, University of Toledo, Toledo, OH, USA, [53]).

### Ethics statement

Mice were maintained according to National Institutes of Health guidelines for the use of experimental animals with the approval of the Indiana University (protocol #10500) and Vanderbilt University Medical Center (protocol #M1600154) Committees for the Use and Care of Animals. Experiments were performed following the United States Public Health Service Policy on Humane Care and Use of Laboratory Animals and the US Animal Welfare Act.

### MRSA skin infection and topical ointment treatment

Mice were infected with MRSA USA300 LAC strain (~3 ×10^6^ colony forming units [CFU]) s.c. in 50 μL phosphate-buffered saline (PBS) as we have previously shown[54]. Lesion and abscess sizes were monitored daily and determined by affected areas calculated using a standard formula for the area: (A = [π/2] × l × w) [55]. The final concentrations of the ointments were as follows: LTB_4_ (33.7 ng– 3.37 × 10^−6^%), U75302 (0.001%), apocynin (16.6%), and mupirocin (0.1%), all in 1 g of petroleum jelly (vehicle control). The treatments were applied to the infected area with a clean cotton swab. Mice were treated once a day throughout infection (ranging from 6 hours to 9 days).

### Skin biopsies and bacterial load

Punch biopsies (8 mm) from noninfected or infected skin were harvested at different time points and used for determining bacterial counts, cytokine production, RNA extraction, cell isolation, histological analyses, and IHC staining [56]. For bacterial load, skin biopsies were collected at days 1 and 9 post-infection, processed and homogenized in TSB medium, and serial dilutions were plated on TSB agar plates and colonies were counted after 18 h at 37°C.

### Primary cell isolation

Resident peritoneal macrophages were isolated using ice-cold PBS as previously described [11, 57].

To isolate bone marrow neutrophils, bone marrow from both tibias and femurs were flushed with PBS with a 26G needle and a 20-mL syringe. Neutrophils were negatively isolated using a MACSxpress Neutrophil Isolation Kit as suggested by the manufacturer (Miltenyi Biotec, Sunnyvale, CA, USA).

### Phagocytosis and bacterial killing assays

Resident peritoneal macrophages and bone marrow-derived neutrophils (2 × 10^5^/well) were plated into two 96-well plates with opaque walls and clear bottoms. Cells were cultured in DMEM and pretreated with 10 μM U75302 (Cayman Chemicals, Ann Arbor, MI) for 30 minutes or 10 nM LTB_4_ for 5 minutes before the addition of MRSA-GFP at a multiplicity of infection of 50:1, as we have previously described [49]. Infected cells were incubated 1 hour to allow phagocytosis, and both plates were washed with warm PBS. The PBS and treated samples were added to the killing plate and incubated for another 2 hours for killing assays. To measure the intensity of GFP fluorescence, extracellular fluorescence was quenched with 50 μL of 500 μg/mL trypan blue, and the GFP fluorescence was quantified using a fluorimeter plate reader. Trypan blue served as blank. A reduction in GFP fluorescence in the killing plate relative to the phagocytosis plate indicated bacterial killing.

### Macrophage depletion studies

To deplete monocytes and macrophages, MMDTR mice were treated with 100 ng of DT once a day for 3 consecutive days before MRSA skin infection [52].

### IVIS Spectrum in vivo imaging

The IVIS SpectrumCT (PerkinElmer, Waltham, MA, USA) instrument was used to image bioluminescent MRSA and macrophages in the skin of the animal.

For bioluminescence imaging, the mice were scanned without emission filtration for 1–4 min/image. Mice were scanned longitudinally once a day for 10 days to monitor the course of MRSA skin infection. For analysis, regions of interest were drawn around each site of infection and the total photon flux (photons/second) was measured. To account for background signal resultant from camera thermal noise and emission scatter, a background region was drawn outside the mouse to determine the background signal of each scan. To establish the relationship between bacterial CFU and background-free total photon flux, bacterial standard curves were prepared and imaged by spotting known bacterial CFU onto TSA plates (for *in vitro* studies) or from subcutaneously infected mice (for *in vivo* studies). By establishing the luminescence and CFU relationship, and fitting these with linear regression, it was possible to quantify the bacterial burden in the skin or each mouse.

For DsRed and mCherry fluorescence imaging, in the presence of bioluminescent MRSA, groups of 4 mice were imaged at 6–8 distinct emission wavelengths over the (excitation: 535, 570, 605) 560-680nm and (excitation: 500, 535, 570) 580-720nm bandwidths, respectively, with an exposure range of 1–5 sec/group. To provide negative controls, WT mice that were DsRed negative (or mCherry negative) with and without bioluminescent MRSA were imaged as above, and served as bioluminescent MRSA and auto-fluorescence controls, respectively. Because the total emission in each mouse is a linear combination of DsRed (or mCherry) fluorescence combined with MRSA bioluminescence, DsRed (or mCherry) the fluorescence emission must be spectrally deconvolved. Individual basis functions for each spectral series were constructed as follows:

$$E_{Auto}\left(\lambda ,i\right)=E_{WT}\left(\lambda \right)$$ (1)

$$E_{BL}\left(\lambda ,i\right)=E_{MRSA}\left(\lambda ,i\right)-E_{Auto}\left(\lambda ,i\right)$$ (2)

$$E_{FL}\left(\lambda ,i\right)=E_{Total}\left(\lambda \right)-E_{BL}\left(\lambda ,i\right)$$ (3)

Where *λ*, *i*, *E*_*Auto*,_ *E*_*WT*_, *E*_*BL*_, *E*_*MRSA*_, *E*_*Total*_, *and E*_*FL*_ are the wavelength, subject, autofluorescence emission, wild-type emission (i.e. no DsRed or mCherry), bioluminescence emission, MRSA emission (i.e. bioluminescence + autofluorescence), total emission (i.e. bioluminescence + autofluorescence + mCherry), and mCherry emission only. Wavelength-dependent spectral basis functions were manually constructed and loaded into LivingImage (PerkinElmer, Waltham, MA, USA), where mCherry spatially dependent signals for each pixel were spectrally deconvolved using a multi-linear least squares approach. Once the images were spectrally decomposed, a region of interest was drawn around the mCherry signal and was the average pixel intensity was reported in total radiant efficiency ([photons/second]/[μW/cm^2^]).

### Two-photon intravital microscopy

Mice were anesthetized with a solution of ketamine/xylazine. A skin flap was created surrounding the infection area, as adapted from [58]. The skin flap was placed in a coverslip-bottomed cell culture dish for imaging and moistened with PBS. The temperature of the mouse was maintained at 36°C with two ReptiTherm pads. Mice were imaged for up to 1 hour. Imaging was performed using an Olympus FV1000-MPE confocal/multiphoton microscope (Olympus, Tokyo, Japan). Analyses were performed using Amaris or FIJI (Image J) tracking software.

### RNA isolation, reverse transcription, and quantitative real-time PCR

Total RNA was isolated from skin biopsies using lysis buffer (RLT; Qiagen, Redwood City, CA, USA) and the cDNA and real-time PCR were performed as previously published [10] using primers indicated in the instructions included with the CFX96 Real-Time PCR Detection System (Bio-Rad, Hercules, CA, USA). Gene-specific primers were purchased from Integrated DNA Technologies (Redwood City, CA, USA). Relative expression was calculated as previously described [10].

### Multiplex bead array (Luminex), ELISA, and EIA assays

Skin biopsy sections were homogenized with a pestle in TNE cell lysis buffer containing 1× protease inhibitor (Sigma-Aldrich, St. Louis, MO, USA) and centrifuged to pellet the cellular debris. Multiplex bead array analysis was performed as suggested by the manufacturer and analyzed using a Bio-Rad Bio-Plex MAGPIX multiplex reader. The Web-based tool Morpheus (https://software.broadinstitute.org/morpheus/) was used to generate heat maps.

LTB_4_ was measured using an EIA kit (Cayman Chemicals, Ann Arbor, MI, USA) following the manufacturer’s protocols. In all the experiments, protein and lipid concentrations were normalized to the total protein concentration in the tissue, as determined by the Bradford method.

### Skin biopsy dissociation for flow cytometry

Skin biopsies were digested with collagenase and processed to obtain a single-cell suspension as previously described [54]. Single cells were stained with the fluorescent antibodies or CellRox for flow cytometry analyses on the BD LSR II flow cytometer (BD Biosciences, San Jose, CA, USA). Analyses were completed using Flow Jo software.

### Histopathology analysis

For histological analysis, 8 μm skin sections were stained with Hematoxylin and eosin or Masson’s trichrome blue stain for capsule visualization [16]. Images of tissue sections were visualized and acquired using the Nikon Eclipse Ci and Nikon Ds-Qi2 (Nikon, Tokyo, Japan).

### Immunohistochemistry of skin biopsies

For histological analyses, the slides from 8 μm paraffin-embedded skin sections were treated with 10% hydrogen peroxide in distilled water to block endogenous peroxidase activity. Slides were blocked with PBS containing 8% serum. Sections were then incubated with anti-Ly6G/C antibody, anti-F4/80, followed by a peroxidase-conjugated secondary antibody, color development, and hematoxylin counterstaining. The 5-LO staining was performed using the Vectastain ABC kit (Vector Labs, Burlingame, CA, USA) as suggested by the manufacturer, and the 5-LO antibody (1:100; Cell Signaling Technology, Danvers, MA, USA) was incubated for 18 hours at room temperature. Images were analyzed using FIJI software. Negative staining controls were generated by omitting the primary antibody. Slides were visualized, and images were acquired using the Nikon Eclipse Ci and Nikon Ds-Qi2 (Nikon, Tokyo, Japan).

### Immunofluorescence staining

The slides from 8 μm sections were incubated in 3% PBS / BSA /Triton X-100 for one hour and incubated with a rabbit anti-5-LO (1:100; Cell Signaling Technology) antibody and goat anti-Ly6G-AlexaFluor488 conjugated (1:100; BD Biosciences) for 1 h followed by sequential washes and incubation with an Alexa-Fluor568 goat anti-rabbit secondary antibody. To image mCherry+ cells in MMDTR mice, biopsy sections were flash frozen, and the slides were prepared on a microtome for immunofluorescence staining. In all circumstances, tissues were stained with 4',6-diamidino-2-phenylindole (DAPI) as a nuclear counterstain. Slides were imaged on a Leica (Wetzlar, Germany) confocal microscope for mCherry, Ly6G, 5-LO and DAPI fluorescence. Slides were visualized and images acquired on the Nikon Eclipse Ci with filters for DAPI, GFP, and Texas Red, using the Nikon Ds-Qi2 camera.

### Statistical analyses

The results are shown as the mean ± SEM and were analyzed using the Prism 5.0 software (GraphPad Software, San Diego, CA, USA). For comparisons between two experimental groups Student’s *t*-test was used, and for comparisons among three or more experimental groups, we used one-way analysis of variance followed by Bonferroni multiple comparison tests. The open software Morpheus heatmap program (https://software.broadinstitute.org/morpheus/) was used to generate heat maps. Values of p < 0.05 were considered significant.

## Supporting information

S1 Fig Representative skin sections of mice infected with MRSA. The indicated animal strains that were infected with MRSA by s.c. injection, followed by treatments with mupirocin and apocynin plus LTB_4_ ointment for 24 h. Skin biopsies were stained with Masson’s Trichrome blue and shown a 100 X magnification. Results are representative of at least 2 individual mice.
(TIF)

S2 Fig LTB_4_/BLT1 signaling is required for collagen formation in the abscess. WT, 5-LO^-/-^ and BLT1^-/-^ mice were infected with MRSA by s.c. injection, followed by treatment with LTB_4_ ointment for 24 h. Skin biopsies were stained with Masson’s Trichrome blue and shown a 40 X (upper) and 400 X (bottom) magnification. Results are representative of 3–5 individual mice.
(TIF)

S3 Fig LTB_4_ controls bacterial clearance, but not cytokine and chemokine production. WT mice were infected with MRSA by s.c. injection, followed by treatment with vehicle control or BLT1 antagonist ointment and 3 h after, skin biopsies collected and the production of **A)** LTB_4_, **B)** CFU counts, **C)** TNF-α production and **D)** CXCL2 were measured using ELISA as described in Material and Methods. Data are the mean ± SEM of 4–5 mice. *p < 0.05 vs. naïve. **E)** H&E stains from mice treated as above and shown at 40 X (upper), and 100 X (bottom) magnification. Images are representative of at least 3 mice/group.
(TIF)

S4 Fig Low infectious dose unveils a role of LTs in bacterial clearance and neutrophil recruitment. WT and 5-LO^-/-^ mice were infected with 5X10^5^ MRSA by s.c. injection and 24 h after, skin biopsies collected and the production of **A)** CFU counts and **B)** CXCL2 were determined as described in Material and Methods. Data are the mean ± SEM of 4–5 mice. *p < 0.05 vs. naïve. **C)** H&E stains from mice treated as above and shown at 40 X (upper), 100 X and 400 X magnification. Images are representative of at least 3 mice/group.
(TIFF)

S5 Fig LTB_4_ topical ointment increases the production of inflammatory mediators during MRSA skin infection. WT mice were infected with MRSA by s.c. injection, followed by treatment with LTB_4_ ointment once a day for 9 days. Biopsies were homogenized and the production of **A)** CXCL1, **B)** CCL4, **C)** MMP8 and **D)** IL-33 were measured using bead array multiplex as described in Material and Methods. Data are the mean ± SEM of 4–5 mice. *p < 0.05 vs. naïve mice and # p < 0.05 vs infected and vehicle-control treated mice.
(TIFF)

S6 Fig Macrophage depletion protocol in MMDTR mice. **A)** DT treatment protocol to deplete macrophages in MMDTR mice. One hundred ng of DT in PBS or PBS was administered via intraperitoneal injections once daily for 3 consecutive days prior to MRSA skin infection. Mice biopsied on day 2 post infection received 100 ng DT on day 1 post infection. **B)** MMDTR mice were infected or not infected with MRSA by s.c. injection. The mCherry fluorescence was measured by the IVIS Spectrum in naïve or day 1 post MRSA skin infected mice. **C)** Biopsy punches were collected at day 1 post MRSA skin infection from PBS-treated or DT-treated MMDTR mice and analyzed for percentage of mCherry^+^ cells by flow cytometry. Data are the mean ± SEM of 3–8 mice. *p < 0.05 vs. PBS-treated.
(TIF)

S7 Fig Macrophage/LTB_4_ axis regulates the production of inflammatory mediators during MRSA skin infection. MMDTR mice were treated with 100 ng of DT or PBS once daily for 3 consecutive days, followed by MRSA skin infection and topical LTB_4_ treatment. 24 hours after infection, biopsies were homogenized and the production of **A)** CCL2, **B)** CCL4, **C)** CXCL2, **D)** VEGF, **E)** IL-1β and **F)** IL-33 were measured using bead array multiplex as described in Material and Methods. **G)** H&E stains from mice treated as above and shown at 10 X (upper) and 400 X magnification. Data are the mean ± SEM of 4–5 mice. *p < 0.05 vs. naïve mice and # p < 0.05 vs infected and vehicle-control treated mice and & p < 0.05 vs DT only treated mice.
(TIF)
