# Supplementary figures and images for: Macrophage-derived LTB4 promotes abscess formation and clearance of Staphylococcus aureus skin infection in mice
Source: PLoS Pathog. 2018 Aug 13;14(8):e1007244. doi: 10.1371/journal.ppat.1007244 (PMC6107286; doi:10.1371/journal.ppat.1007244)

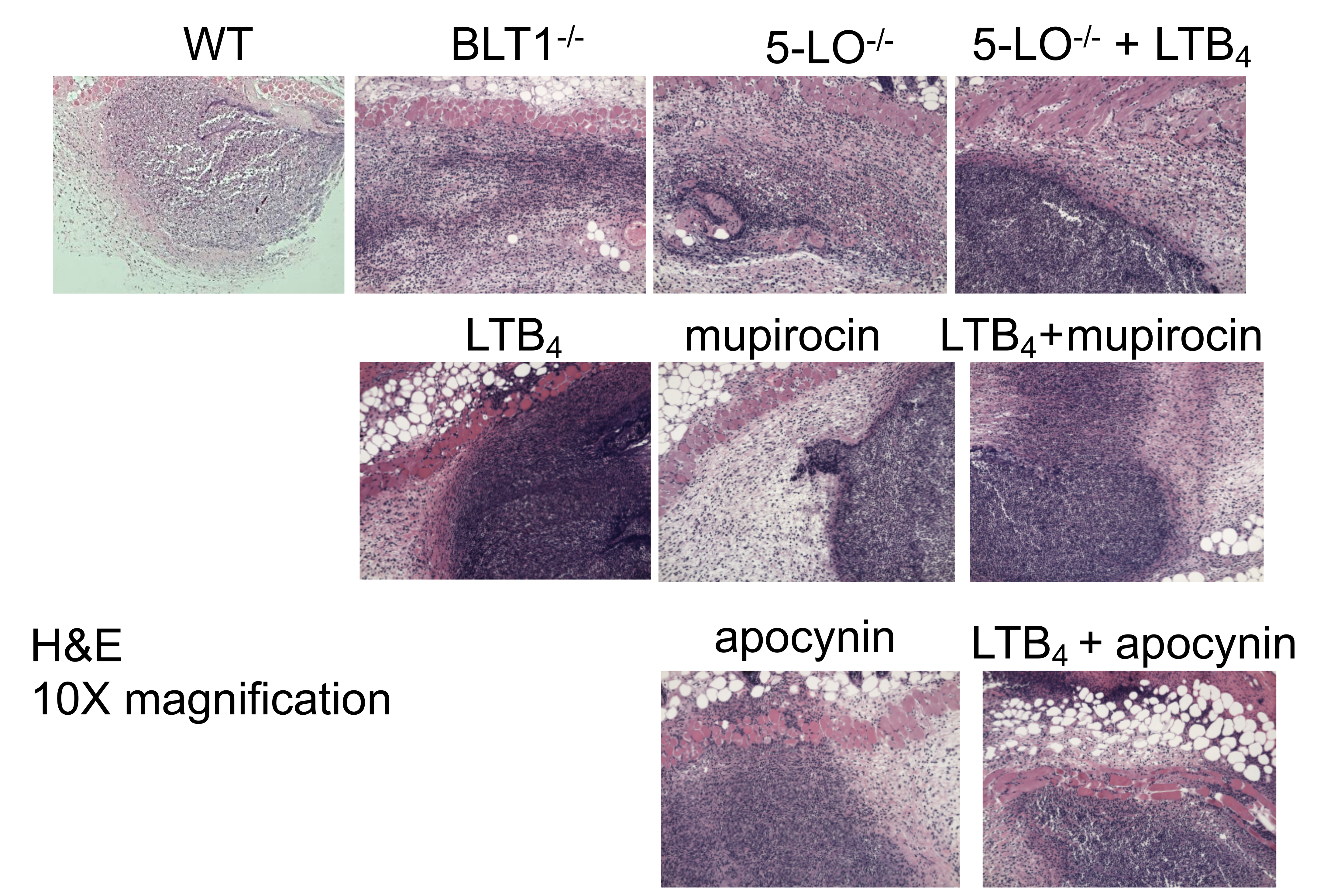

Supplement: S1 Fig — The indicated animal strains that were infected with MRSA by s.c. injection, followed by treatments with mupirocin and apocynin plus LTB4 ointment for 24 h. Skin biopsies were stained with Masson’s Trichrome blue and shown a 100 X magnification. Results are representative of at least 2 individual mice. (TIF) [file ppat.1007244.s001.tif]

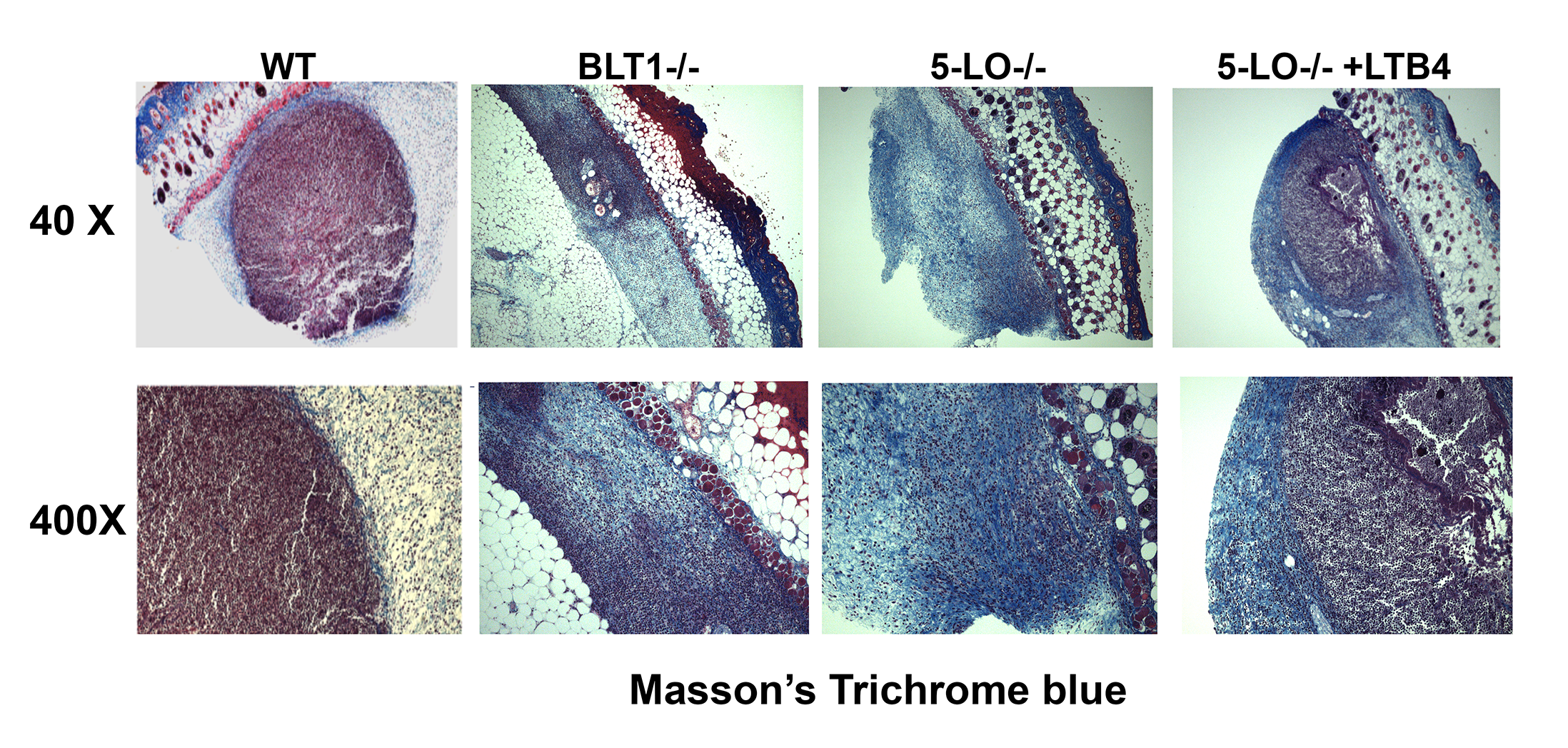

Supplement: S2 Fig — WT, 5-LO-/- and BLT1-/- mice were infected with MRSA by s.c. injection, followed by treatment with LTB4 ointment for 24 h. Skin biopsies were stained with Masson’s Trichrome blue and shown a 40 X (upper) and 400 X (bottom) magnification. Results are representative of 3–5 individual mice. (TIF) [file ppat.1007244.s002.tif]

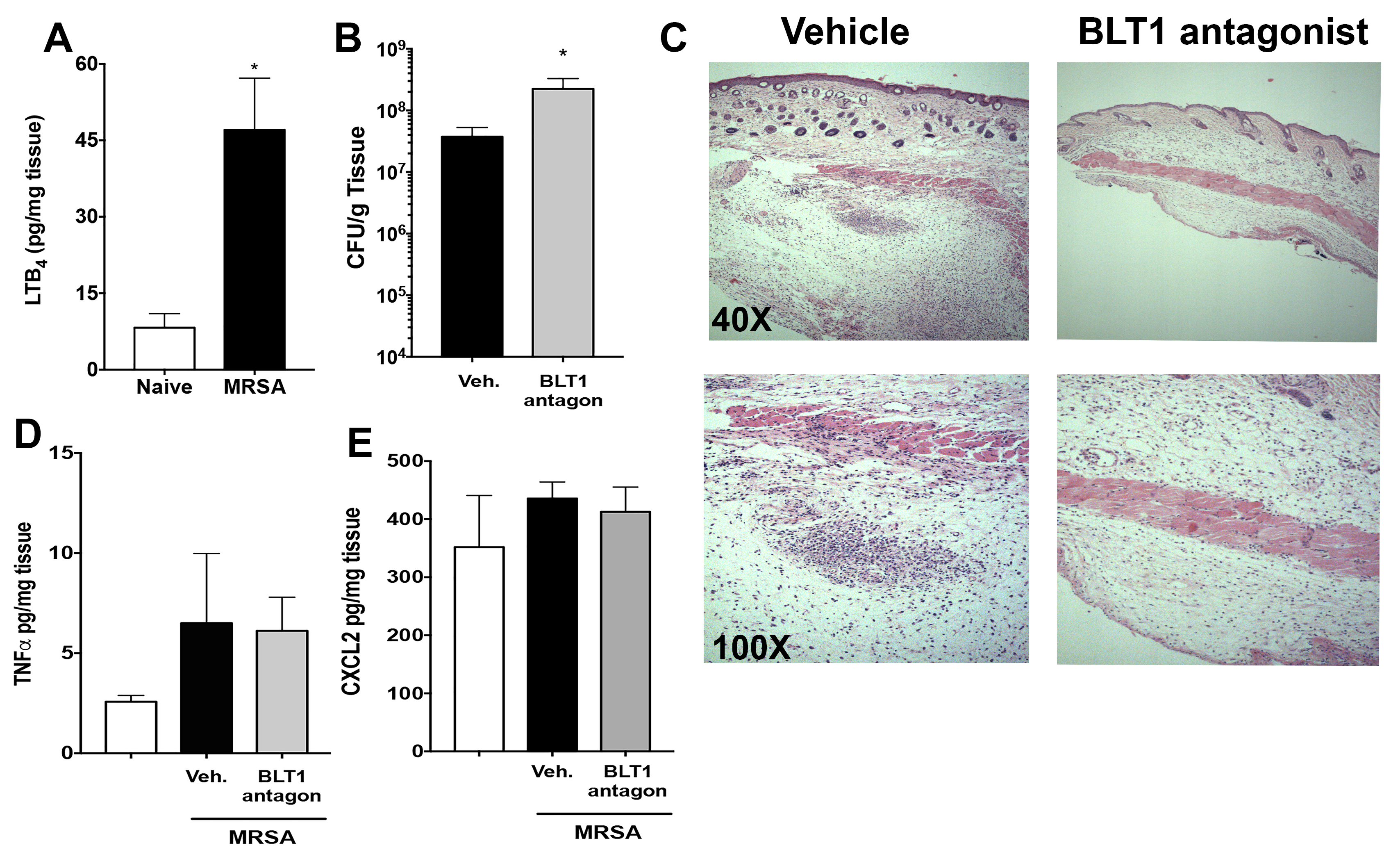

Supplement: S3 Fig — WT mice were infected with MRSA by s.c. injection, followed by treatment with vehicle control or BLT1 antagonist ointment and 3 h after, skin biopsies collected and the production of A) LTB4, B) CFU counts, C) TNF-α production and D) CXCL2 were measured using ELISA as described in Material and Methods. Data are the mean ± SEM of 4–5 mice. *p < 0.05 vs. naïve. E) H&E stains from mice treated as above and shown at 40 X (upper), and 100 X (bottom) magnification. Images are representative of at least 3 mice/group. (TIF) [file ppat.1007244.s003.tif]

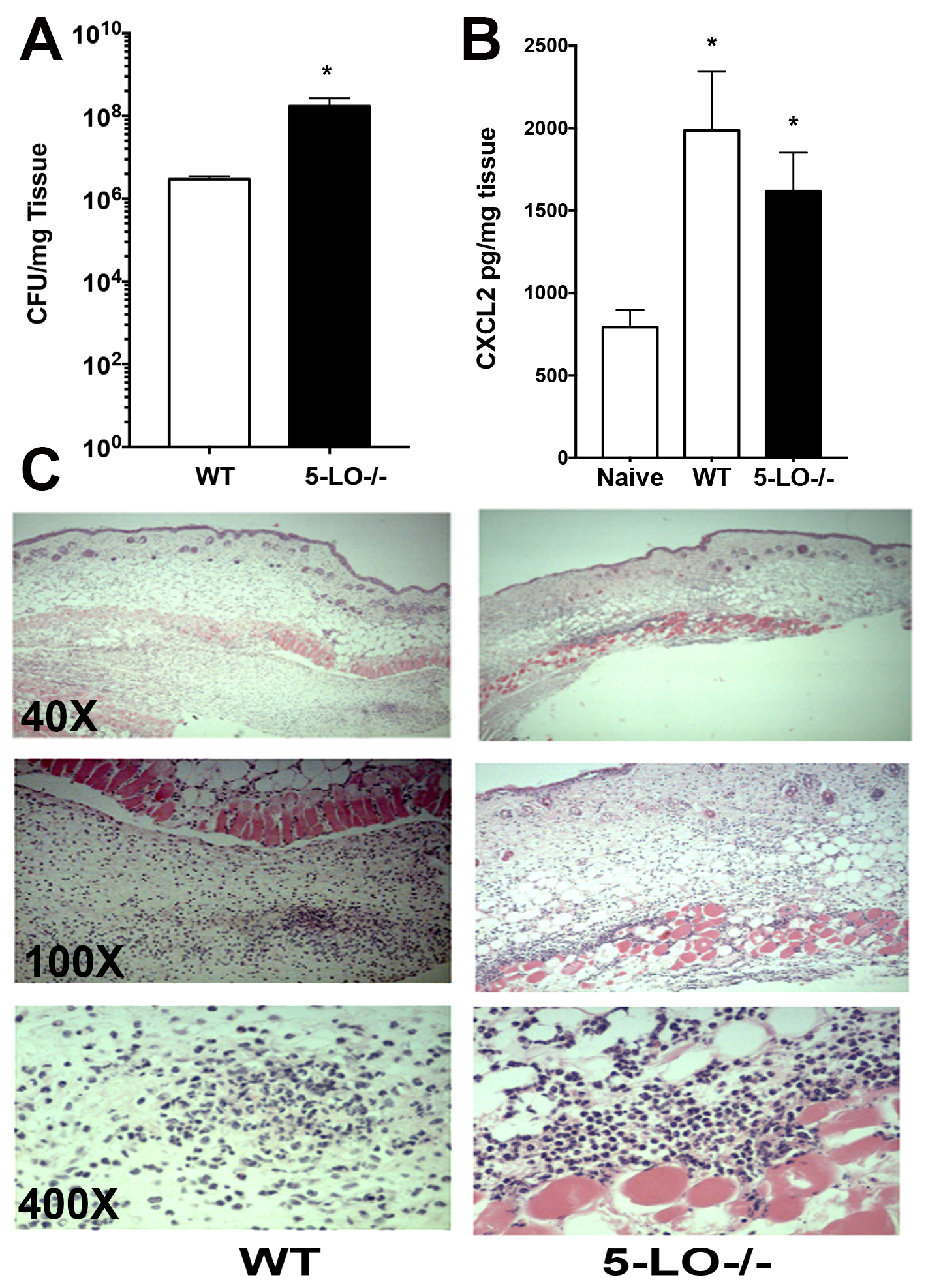

Supplement: S4 Fig — WT and 5-LO-/- mice were infected with 5X105 MRSA by s.c. injection and 24 h after, skin biopsies collected and the production of A) CFU counts and B) CXCL2 were determined as described in Material and Methods. Data are the mean ± SEM of 4–5 mice. *p < 0.05 vs. naïve. C) H&E stains from mice treated as above and shown at 40 X (upper), 100 X and 400 X magnification. Images are representative of at least 3 mice/group. (TIFF) [file ppat.1007244.s004.tiff]

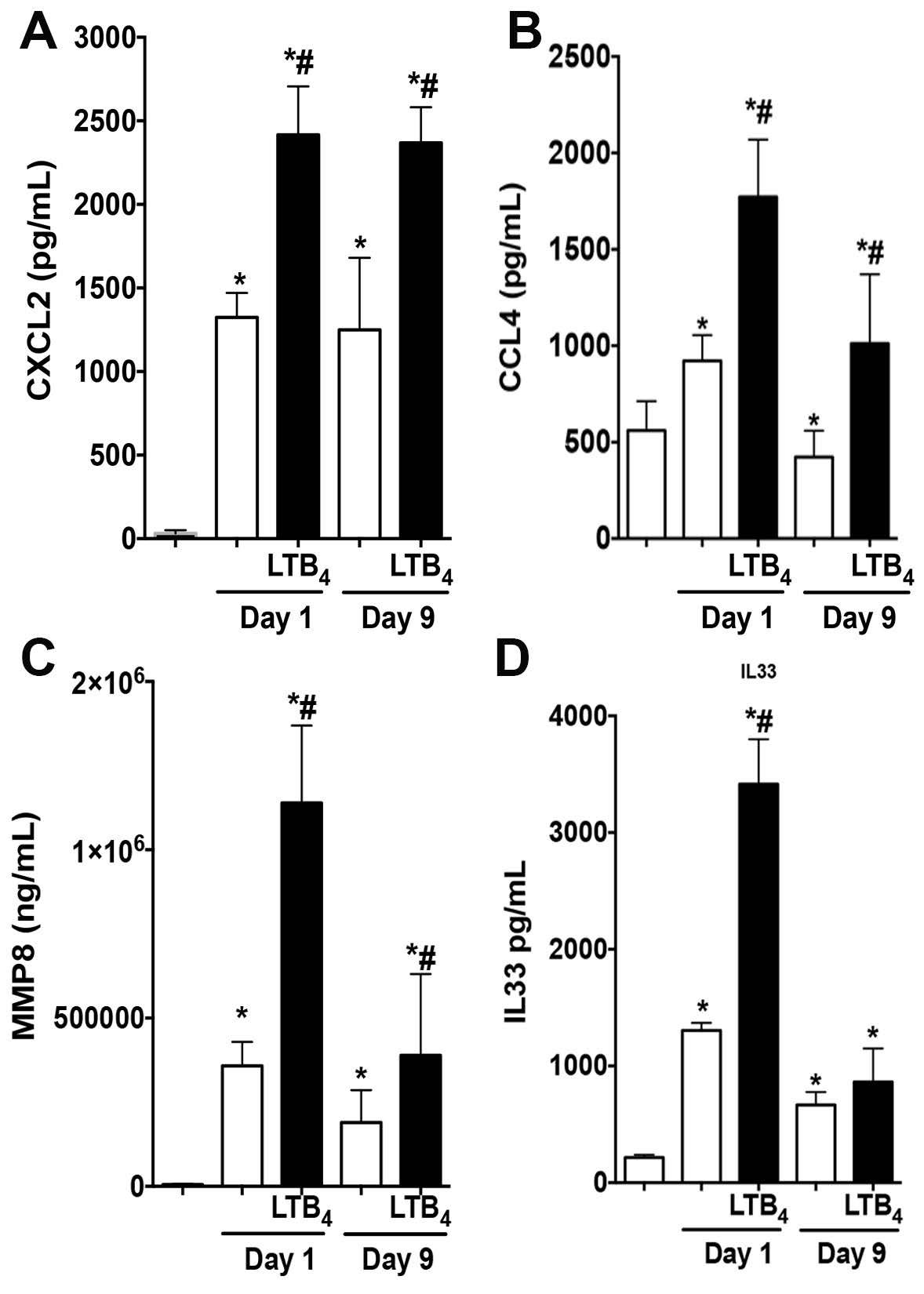

Supplement: S5 Fig — WT mice were infected with MRSA by s.c. injection, followed by treatment with LTB4 ointment once a day for 9 days. Biopsies were homogenized and the production of A) CXCL1, B) CCL4, C) MMP8 and D) IL-33 were measured using bead array multiplex as described in Material and Methods. Data are the mean ± SEM of 4–5 mice. *p < 0.05 vs. naïve mice and # p < 0.05 vs infected and vehicle-control treated mice. (TIFF) [file ppat.1007244.s005.tiff]

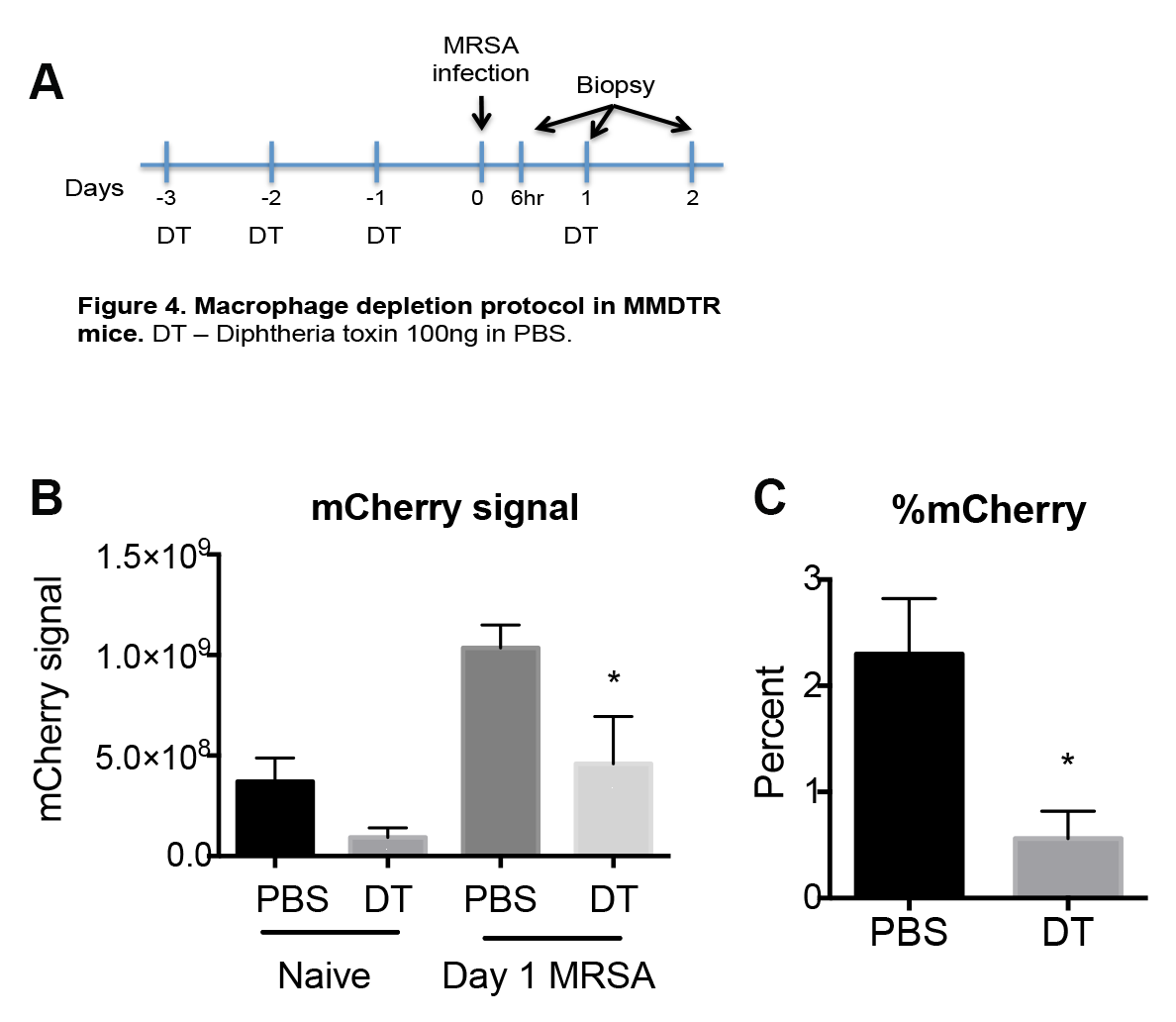

Supplement: S6 Fig — A) DT treatment protocol to deplete macrophages in MMDTR mice. One hundred ng of DT in PBS or PBS was administered via intraperitoneal injections once daily for 3 consecutive days prior to MRSA skin infection. Mice biopsied on day 2 post infection received 100 ng DT on day 1 post infection. B) MMDTR mice were infected or not infected with MRSA by s.c. injection. The mCherry fluorescence was measured by the IVIS Spectrum in naïve or day 1 post MRSA skin infected mice. C) Biopsy punches were collected at day 1 post MRSA skin infection from PBS-treated or DT-treated MMDTR mice and analyzed for percentage of mCherry+ cells by flow cytometry. Data are the mean ± SEM of 3–8 mice. *p < 0.05 vs. PBS-treated. (TIF) [file ppat.1007244.s006.tif]

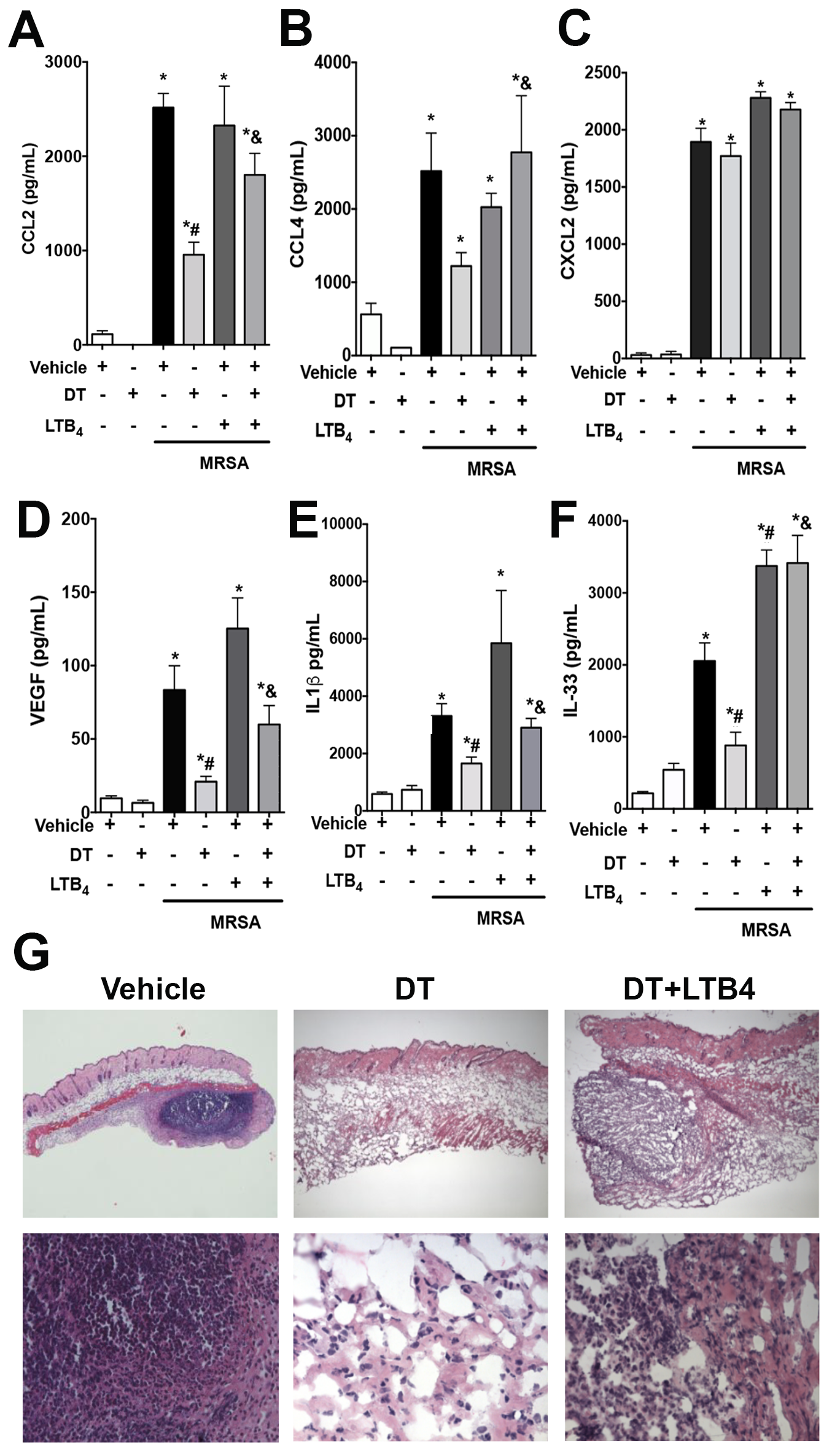

Supplement: S7 Fig — MMDTR mice were treated with 100 ng of DT or PBS once daily for 3 consecutive days, followed by MRSA skin infection and topical LTB4 treatment. 24 hours after infection, biopsies were homogenized and the production of A) CCL2, B) CCL4, C) CXCL2, D) VEGF, E) IL-1β and F) IL-33 were measured using bead array multiplex as described in Material and Methods. G) H&E stains from mice treated as above and shown at 10 X (upper) and 400 X magnification. Data are the mean ± SEM of 4–5 mice. *p < 0.05 vs. naïve mice and # p < 0.05 vs infected and vehicle-control treated mice and & p < 0.05 vs DT only treated mice. (TIF) [file ppat.1007244.s007.tif]
